# Cutmarked bone of drought-tolerant extinct megafauna deposited with traces of fire, human foraging, and introduced animals in SW Madagascar

**DOI:** 10.1038/s41598-022-22980-w

**Published:** 2022-11-22

**Authors:** Sean W. Hixon, Alejandra I. Domic, Kristina G. Douglass, Patrick Roberts, Laurie Eccles, Michael Buckley, Sarah Ivory, Sarah Noe, Douglas J. Kennett

**Affiliations:** 1 Department of Archaeology, Max Planck Institute for Geoanthropology, Jena, Germany; 2 Department of Geosciences and the Earth and Environmental Systems Institute, Pennsylvania State University, University Park, PA USA; 3 Department of Anthropology, Pennsylvania State University, University Park, PA USA; 4 Climate School, Columbia University, New York, NY USA; 5 School of Natural Sciences, Manchester Institute of Biotechnology, The University of Manchester, Manchester, M1 7DN UK; 6 Department of Anthropology, University of California, Santa Barbara, CA USA; 7 isoTROPIC Research Group, Max Planck Institute for Geoanthropology, Jena, Germany

**Keywords:** Palaeoecology, Palaeoecology, Archaeology

## Abstract

People could have hunted Madagascar’s megafauna to extinction, particularly when introduced taxa and drought exacerbated the effects of predation. However, such explanations are difficult to test due to the scarcity of individual sites with unambiguous traces of humans, introduced taxa, and endemic megaherbivores. We excavated three coastal ponds in arid SW Madagascar and present a unique combination of traces of human activity (modified pygmy hippo bone, processed estuarine shell and fish bone, and charcoal), along with bones of extinct megafauna (giant tortoises, pygmy hippos, and elephant birds), extirpated fauna (e.g., crocodiles), and introduced vertebrates (e.g., zebu cattle). The disappearance of megafauna from the study sites at ~ 1000 years ago followed a relatively arid interval and closely coincides with increasingly frequent traces of human foraging, fire, and pastoralism. Our analyses fail to document drought-associated extirpation or multiple millennia of megafauna hunting and suggest that a late combination of hunting, forest clearance, and pastoralism drove extirpations.

## Introduction

A diversity of large animals endemic to Madagascar disappeared during the last millennium, including elephant birds, giant lemurs, pygmy hippos, and giant tortoises^1^. Early to mid-Holocene evidence of the timing of human arrival is still the subject of much debate^2–5^ but raises the possibility that there were millennia of coexistence between humans and endemic megafauna on the island^6^. Extended coexistence would be noteworthy given that it (1) is rare in insular settings^7^, and (2) requires explanations of relatively late extinctions to involve more than the mere presence of human hunters on the landscape^8,9^. Nevertheless, the scarcity of sites with clear traces of people and past megafauna means that the extent and nature of human association with endemic megafauna remains poorly known.

A multitude of factors (e.g., deforestation, drought, disease, and competition) could have exacerbated the impact of recent human hunting on megafauna^10^, with evidence of all documented from the past thousand years^4,11–13^. For example, sedimentary charcoal and pollen records include increasingly frequent indications of fire and forest clearance during the past millennium^14,15^. Directly dated remains of livestock [e.g., cattle^9^] and crops [e.g., rice^16^] from ~ 1 kya reflect the arrival of pastoralism and farming, which supported people in widely settling and modifying the island. Although poorly characterized, the relative tolerance of introduced and endemic taxa to particular adaptive challenges (e.g., water scarcity) is critically important given that it influences the outcome of interspecific interactions^17,18^. These tolerances can be inferred partly by recognizing changes in a taxon’s effective population size, habitat use, and geographic distribution that coincide with past stressors over millennia^19–21^.

**Figure 1:**
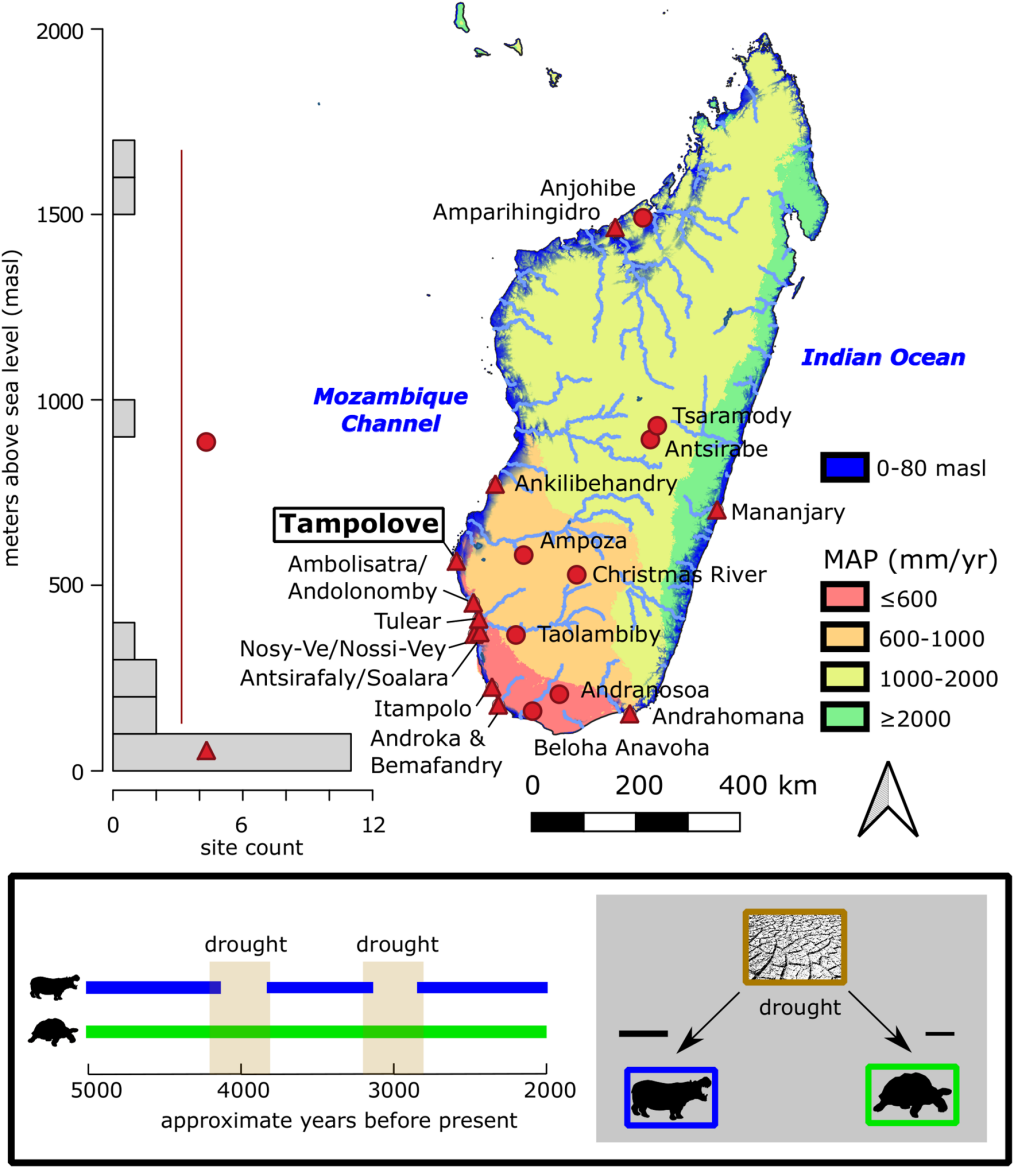
Geographic context of study area (Tampolove), and concept of drought tolerance based on past occurrence data. Points mark the sites that include ^14^C dated collagen from extinct pygmy hippos (*Hippopotamus* spp.) and giant tortoises (*Aldabrachelys* spp.). Most sites are in the coastal lowlands (triangles), where changes in both climate and relative sea level can contribute to drought. At arid coastal sites, we would expect a relatively drought sensitive animal (e.g., hippos) to be extirpated during past drought as ranges contract up comparably wet inland drainages. Note that rainfall data reported as mean annual precipitation are taken from WorldClim 2.1^35^ and that map was generated in QGIS (version 3.10.2, www.qgis.org).

Water is a limiting resource in southern Madagascar and constrains the extent of forest habitat elsewhere on the island. While NW Madagascar was likely generally more mesic during the Holocene^22–24^, speleothem records from SW Madagascar suggest that the early Holocene was arid^25,26^ and that the mid to late Holocene was punctuated by arid intervals^27,28^. A marine transgression and subsequent regression during the late Holocene can also account for lowering coastal water tables^29,30^. Though water scarcity is a long-hypothesized driver of megafaunal extinction^31^, proxies of habitat aridity suggest that endemic animals had some level of drought tolerance^32,33^. We expect the geographic range of a drought sensitive animal to shrink in tandem with wet habitat. At a regional scale on Madagascar, this would first involve abandonment of the sites in the semi-arid coastal south (Fig. 1^26^), which has sporadic, highly seasonal rainfall^34^, receives river water (often seasonal) from the interior, and includes a series of low elevation ponds that are prone to salinization during climate drying and marine transgression followed by regression.

Here, we directly radiocarbon (^14^C) date a rare combination of traces of human activity, endemic megafauna, and human-introduced species excavated from several coastal ponds in SW Madagascar to establish a chronology for when each of these groups was present in the area since the middle Holocene. Such a chronology, when coupled with paleoclimate data and local stratigraphic records sensitive to fire, herbivore abundance, and water level can test key implications of the ideas that endemic megaherbivores were sensitive to (1) drought, (2) hunting, and (3) hunting in the presence of introduced domesticated species such as cattle and dogs.

**Figure 2:**
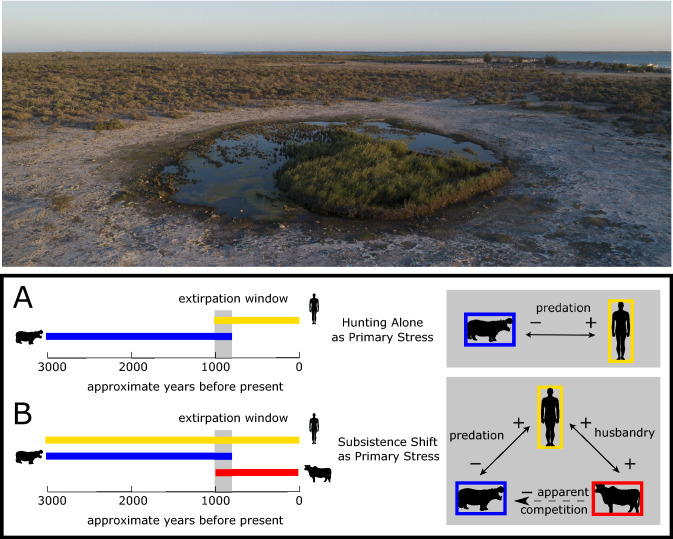
Drone photo of excavated pond TAMP near Tampolove, and possible chronologies underlying two conceptual scenarios (**A** and **B**) for human contributions to megafaunal decline. Freshwater ponds such as Tampolove are points of attraction for animals in arid SW Madagascar, and their sediments include bones that span recent millennia. If humans directly contributed to megafaunal extirpation through hunting (**A**), then we expect the local disappearance of megafaunal bone to coincide with the earliest traces of human activity. If a shift in human food production contributed to extirpation (**B**), then we expect the disappearance of megafaunal bone to coincide instead with traces of this past shift and not necessarily with the earliest traces of local human activity.

1. If some megaherbivores (e.g., pygmy hippos) were more sensitive to water scarcity relative to others (e.g., giant tortoises), then we would expect the sensitive taxa to disappear from the vicinity of ephemeral coastal ponds during past arid intervals (Fig. 1). Persistence of megafauna in place during past dry intervals would suggest either that these animals tolerated water scarcity or that these droughts had minor impacts on local water availability.
2. If megafauna were sensitive merely to the presence of human hunters on the landscape, then we would expect extirpation to have shortly followed the arrival of people (Fig. 2A).
3. If megafauna were sensitive to human hunters only after human populations expanded in tandem with the spread of farming and animal husbandry, then we would expect some period of coexistence of human foragers and megafauna in the absence of a suite of introduced species (Fig. 2B).

## Results

Each sedimentary sequence from the three excavated ponds (Tampolove [TAMP], Ankatoke [ANKA], and Andranobe [ANDR]) includes a layer of clay (defined as zone 2), which separates the surface soil formation (zone 1) from the underlying fossiliferous muddy sand and bedrock (zone 3, Figs. S4–S7 & S9). Details regarding the composition of this sediment and its microfossils are given in *Appendix-Results-Excavation* (Figs. S9–S12).

### Subfossils and chronology

Coastal survey recovered mostly zebu bones on exposed sandy surfaces, some pygmy hippo and giant tortoise bones on the margins of shallow ponds, and giant tortoise carapace under overhanging limestone outcrops (*Appendix-Results-Survey*, Fig. S3). A high proportion of surface bone failed ^14^C analysis (~ 55%, Table S1), yet the successfully analyzed specimens (n = 8) span up to 3390–3220 calibrated years before present (cal BP, PSUAMS 8681, 3150 ± 15 ^14^C BP, a hippo molar). Pond deposits that are relatively deep include bones that cover a relatively long period of time (Figs. S14–S16, Dataset S6). This span ranges from ~ 6000 years at TAMP (~ 120 cm deep) to ~ 2500 years at ANDR (~ 100 cm deep), with the oldest bones present in the fossiliferous sedimentary zone 3 and scarce bones in the overlying clay (zone 2).

#### Zone 3

Most bones in this layer are relatively intact and include readily identifiable pygmy hippo long bones and cranial fragments (e.g., Fig. S13a,f), giant tortoise carapace and plastron fragments (Fig. S13d), ratite eggshell and long bones (Fig. S13c,m), and crocodile scutes, cranial fragments, and teeth (Fig. S13b). Scarce bones of a duck (genus *Anas*) were recovered at ANDR. Remains of subfossil lemurs were scarce or absent, but they may be represented by an unknown type of bone fragment identified through protein fingerprinting (ANDR-1-5-55, Dataset S3). The widespread success of collagen extraction from these bones attests to the excellent preservation of organics in this zone. ANKA also includes keratin (mostly in the form of crocodile claws, e.g., Fig. S13i), as well as two rounded agates found associated with ratite eggshell (Fig. S13m).

Remains of a juvenile pygmy hippo were recovered from both TAMP and ANDR (a femur and tibia, respectively, Dataset S3). The epiphyses of some of the pygmy hippo long bones have gnaw marks (Fig. S13f), and none of the bones include chop marks. In association with these bones towards the top of this zone are some large (> 1 cm diameter) charcoal fragments and scarce bones of bushpig (Fig. S13k) and zebu (Fig. S13e). Protein fingerprinting identified a screened fragment of a non-zebu bovid in ANKA zone 3 and confirmed that a tentatively identified bushpig canine fragment (ANKA 1-4-151) belonged to a hippo. This zone at TAMP and ANDR also includes occasional mangrove whelk (*Terebralia palustris*) shells (Fig. S13g). These whelks currently live at least ~ 500 m distant from these ponds, and whelk shells at ANDR each have an irregular hole above the operculum.

The span of time represented by bones in zone 3 ranges up to ~ 4000 years (~ 6000–2000 cal BP at TAMP, Fig. S14). Confirmed introduced animal bones from zone 3 failed direct ^14^C analysis. There are multiple examples of directly ^14^C-dated bone in close stratigraphic association that nonetheless differ in age by > 1000 years, and there are a couple of examples of bones from the same individual that are separated stratigraphically. For example, two giant tortoise carapace and plastron fragments from TAMP that have indistinguishable ^14^C ages are separated by 22 cm of sediment (PSUAMS 8670 comes from 112 cm depth, and PSUAMS 8668 comes from 90 cm depth).

**Figure 3:**
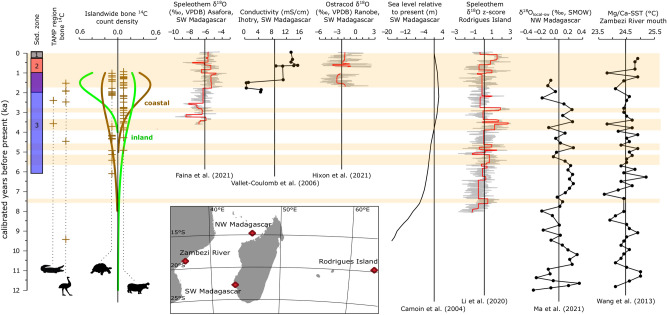
Occurrence of Tampolove fauna on a scale of absolute time relative to local sedimentary zones (Fig. S9), approximate changes in relative sea level on Madagascar, and regional paleoclimate records from points marked in the inset map. Islandwide bone collagen ^14^C date count densities are given for giant tortoises and hippos and are separated according to whether data come from coastal lowlands or inland sites (Fig. 1). Bayesian change point analysis (BCPA) in the Asafora, Ranobe, and Rodrigues records (red lines) identify intervals that can be approximated reasonably well by a single mean, and the overall means of these records are given by black vertical lines. Intervals with above average values highlighted by BCPA in at least one record (indicative of relatively arid conditions) are highlighted with brown horizontal bars for reference. Note that the plot includes two pygmy hippos and two giant tortoises that are likely duplicate specimens.

Although ANKA produced what is thus far the oldest directly ^14^C dated pygmy hippo bone from a coastal subfossil site (PSUAMS 9383, 4380 ± 25 BP, 5030–4840 cal BP), the mean calibrated age of hippos from the Tampolove excavations (n = 11, x̄ = 2858 cal BP, SD = 972 yr) is significantly less than that of the giant tortoises (n = 9, x̄ = 4582 cal BP, SD = 705 yr, t(18) = − 4.4, *p* < 0.001). The success rates of directly ^14^C-dating pygmy hippo versus giant tortoise remains in zone 3 sediment are comparable (10/13 and 9/13, respectively). The pattern of relatively old giant tortoise remains at coastal subfossil sites is conserved through the island-wide review of ^14^C data (Fig. 3). However, this pattern is inverted at inland sites, and the median calibrated age of hippos from inland sites (n = 57, m = 2595 cal BP) is significantly greater than the median calibrated age of hippos from coastal lowland sites (n = 68, m = 1600 cal BP, Mann–Whitney U = 1459, *p* = 0.02). The limited timespan of deposition of pygmy hippo bone at low coastal sites cannot be explained by sampling bias as more pygmy hippo bones from low coastal sites have been directly ^14^C dated (n = 69, as opposed to n = 57 at inland sites), and relatively more low coastal sites have been sampled (n = 10, as opposed to n = 8).

Many animals that lived near Tampolove during the deposition of zone 3 sediments between ~ 5000 and 2000 cal BP could have experienced rising sea levels^30^ and a wetting trend marked by the resumption of speleothem formation in multiple caves in SW Madagascar by ~ 3500 cal BP^25–27^. Directly ^14^C dated remains of both giant tortoises (n = 10) and pygmy hippos (n = 5) attest to the fact that these taxa persisted locally before this wetting trend (Fig. 3). The scarcity of mid-Holocene climate records from SW Madagascar and evidence for asynchronous climate change between the northern and southern parts of the island^27^ complicate inference of mid-Holocene climate change in the region. However, speleothem records from Rodrigues Island (~ 2100 km distant from our study site yet generally coherent with records from NW Madagascar) suggest that “megadroughts” affected at least parts of the SW Indian Ocean during approximately 4760–4600 cal BP and 3880–3280 cal BP^28^. Previously published ^14^C data from bones of pygmy hippos collected from low coastal sites outside of the Tampolove area (n = 41) do not span these intervals, with the exception of a hippo from Nosy-Ve/Nossi-Vey (PSUAMS 5424, 4125 ± 25 ^14^C BP, 4810–4440 cal BP). However, the longest possibly relatively arid interval (~ 600 years, 3880–3280 cal BP) likely encompassed the death of six analyzed individuals: 4 hippos, 1 giant tortoise, and 1 crocodile (with calibrated 95% intervals that span all of the ~ 600-year dry interval).

#### Zone 2

**Figure 4:**
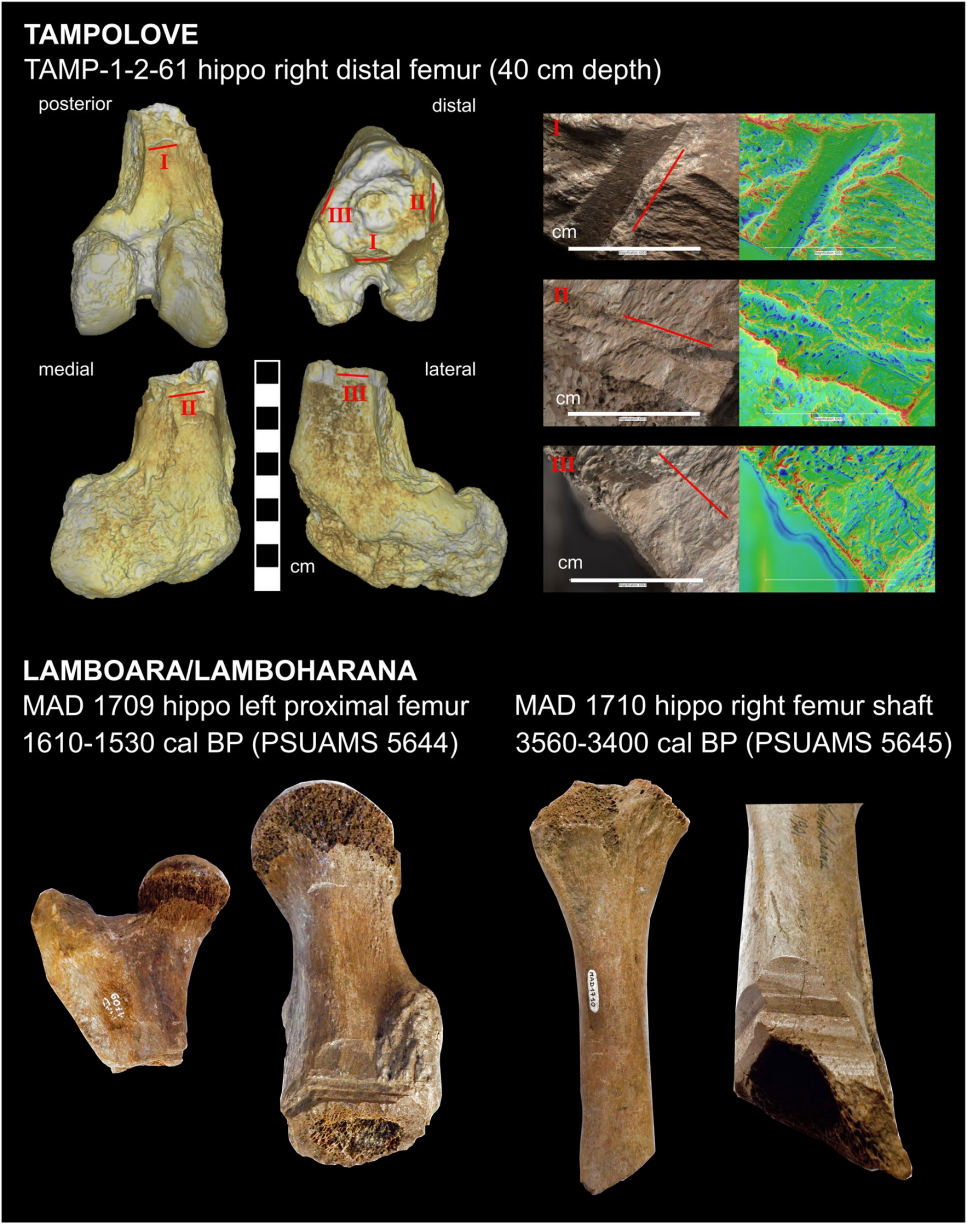
Cutmarked pygmy hippo femur recovered from Tampolove during recent excavation at ~ 40 cm depth (TAMP-1-2-61, above), and previously-recovered and directly ^14^C-dated (~ 3500 and 1600 cal BP^37^) cutmarked pygmy hippo femora from the nearby site of Lamboara/Lamboharana that are currently housed in the National Museum of Natural History in Paris (MAD 1709 & MAD 1710, below). Four views highlight three locations of cutmarks on the broken shaft of TAMP-1-2-61, and the inset frames show 20 × magnification of these areas, with corresponding orientations given by red lines. Note that the false color insets of TAMP-1-2-61 are meant to highlight linear edges and crevices, and the overview photos of all three femur fragments are on the same scale.

Bones in this layer are relatively scarce, fragmentary, and chalky, yet readily identifiable fragments of hippo (Fig. S13h), giant tortoise, crocodile, and zebu cattle are present. At TAMP, protein fingerprinting identified a small fragment of a pygmy hippo long bone as shallow as 24 cm depth (TAMP 1-2-48). TAMP zone 2 includes both a chopped distal fragment of a pygmy hippo right femur (Fig. 4) and an associated scatter of marine fish bones (cranial fragments, vertebrae ~ 1 cm in diameter, and spines), one of which (a vertebra) includes a chop mark (Fig. S13j). Both the chopped pygmy hippo fragment and associated fish bones failed ^14^C analysis due to the exceptionally poor preservation of bone collagen. Charcoal fragments with provenience in zone 2 come from only ANKA and ANDR, and shells of the mangrove whelk are relatively abundant in ANDR zone 2.

The only directly dated collagen from zone 2 (extracted from a fragmentary pygmy hippo molar from ANKA, 55 cm depth, PSUAMS 8733, 3555 ± 20 ^14^C BP, 3880–3700 cal BP, Fig. S15) is > 2000 years older than a closely associated charcoal sample (38 cm depth, PSUAMS 8849, 575 ± 30 ^14^C BP, 630–510 cal BP), which makes this molar comparable in age to bone from zone 3. Consequently, the youngest directly ^14^C-dated ancient bone from the Tampolove excavations comes from the lowermost zone 3: a pygmy hippo’s vertebra recovered at 90 cm depth at TAMP (PSUAMS 8730, 1865 ± 15 ^14^C BP, 1819–1705 cal BP). Though poorly constrained in time, the deposition of zone 2 sediment came sometime within the past two millennia, which witnessed marine regression and dry intervals recorded in both the δ^18^O record of a nearby speleothem^27^ and the salinization of a nearby pan^36^. Previously directly ^14^C-dated bone collected around Tampolove attests to the local persistence of at least pygmy hippos and giant tortoises until the start of the last millennium (n = 15), and an atlas from Lamboara/Lamboharana is in fact the most recent confidently dated pygmy hippo bone from the island (PSUAMS 5629, 1100 ± 15 ^14^C BP, 980–930 cal BP).

#### Zone 1

A fragment of iron (from TAMP, 16 cm depth) and sparse ceramic fragments (from ANKA, 3 & 9 cm depth) are present only in zone 1, and three ^14^C dates from TAMP and ANKA suggest that these specimens span the past ~ 200 years (Figs. S14–S15).

### Charcoal

**Figure 5:**
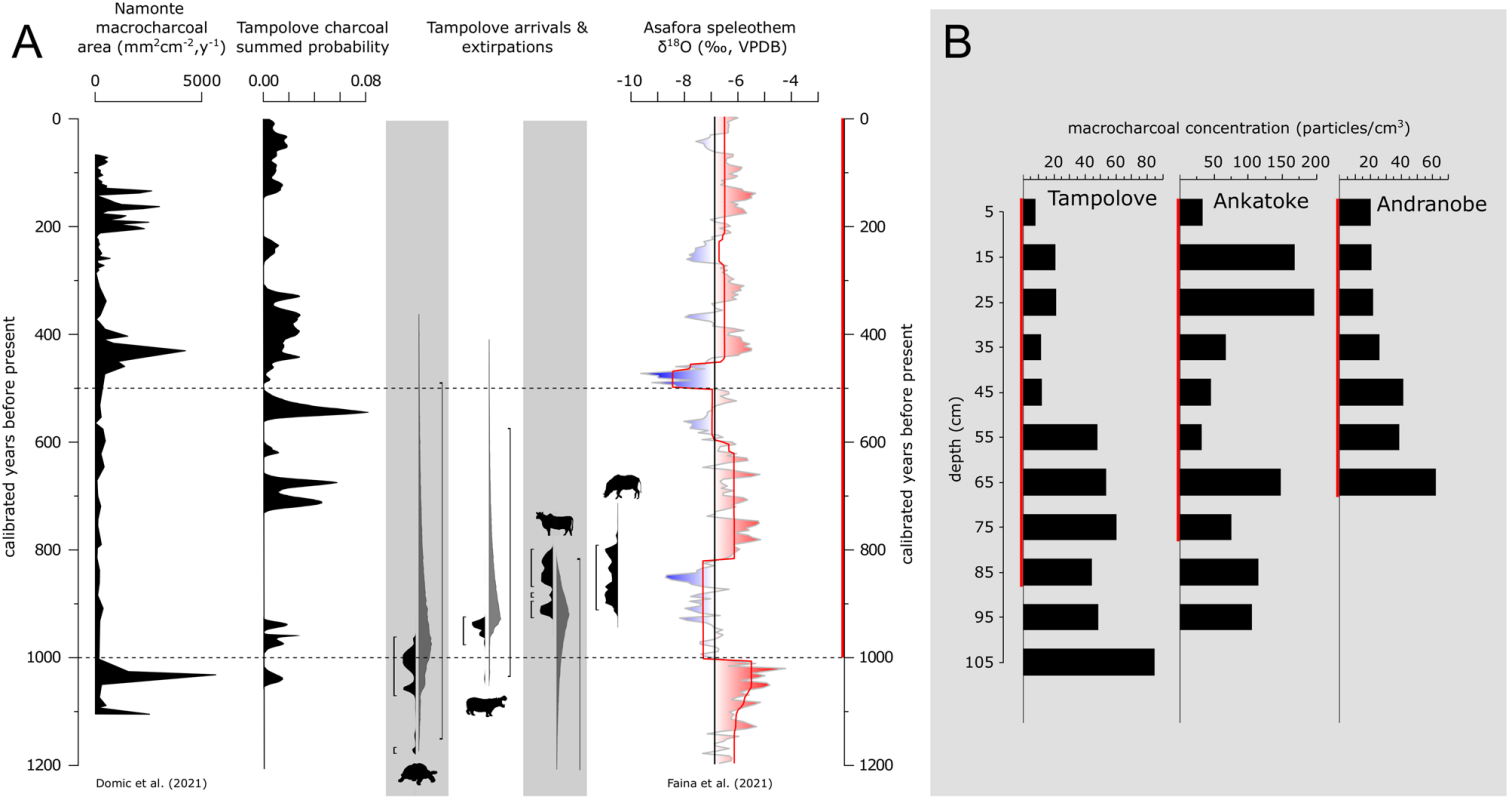
Records of fire, drought, and faunal turnover from the vicinity of Tampolove within the past 1200 years, with dashed horizontal lines for reference (5A), and macrocharcoal concentrations from the excavated ponds, with depth intervals containing directly ^14^C-dated charcoal that spans the past millennium marked in red (5B). The past 1200 years includes the entire summed calibrated distribution of the 10 directly dated prebomb charcoal fragments from the Tampolove excavations. The calibrated probability distributions associated with the latest dates from endemic megafauna bone (giant tortoises and pygmy hippos) and earliest dates from introduced animal bone (zebu cattle and bushpigs) are shown as black distributions, and 95% of each distribution is bracketed. Considering directly dated remains within the past 4 ka from hippos (n = 26), giant tortoises (n = 18), and zebu (n = 9) and the assumption that bones were deposited uniformly over time, the grey distributions and bracketed 95% credible intervals give estimates of extirpation and arrival times. As in Fig. 3, the red line on the Asafora record follows from BCPA.

The directly ^14^C dated charcoal spans all three stratigraphic zones yet consistently dates to the past millennium (Figs. S14–16). Multiple charcoal samples from different excavated ponds have practically indistinguishable ^14^C ages (Table S2), and much of the charcoal from Tampolove formed during peaks in the deposition of macrocharcoal at nearby Namonte (17 km distant; Fig. 5A). The onset of directly ^14^C-dated charcoal deposition approximately coincides with a decrease in Asafora speleothem δ^18^O values and with multiple directly ^14^C-dated first and final local occurrences of large animals. While directly ^14^C dated charcoal is limited to the past millennium, microcharcoal particles were abundant in all TAMP sediment samples (x̄ ± SD = 2.0 × 10^6^ ± 2.8 × 10^6^ particles). Additionally, microcharcoal is relatively abundant near the bottom of TAMP and ANKA, which contains bones that span ~ 6000–2000 cal BP (Fig. 5B).

## Discussion

The rare combination of archaeological, paleontological, and paleoenvironmental records from the vicinity of Tampolove provides an unparalleled opportunity to develop more nuanced understandings of potential megafaunal extinction drivers on Madagascar. We report coincident shifts in the middle Holocene geographic ranges of megafauna and water availability, but there is little evidence that late Holocene aridification alone drove the local disappearance of megafauna. Moreover, while we report cutmarked bone of extinct megafauna associated with other traces of human activity, the stratigraphic context and quality of the cutmarks urge caution in interpreting it as direct evidence of past human hunting. Indeed, rather than unequivocal evidence of climate change or hunting resulting in Madagascan megafaunal demise, research at Tampolove, in the context of island-wide paleoenvironmental evidence, highlights that the disappearance of megafauna closely coincided with deforestation and the spread of pastoralism (Fig. 2B).

**Figure 6:**
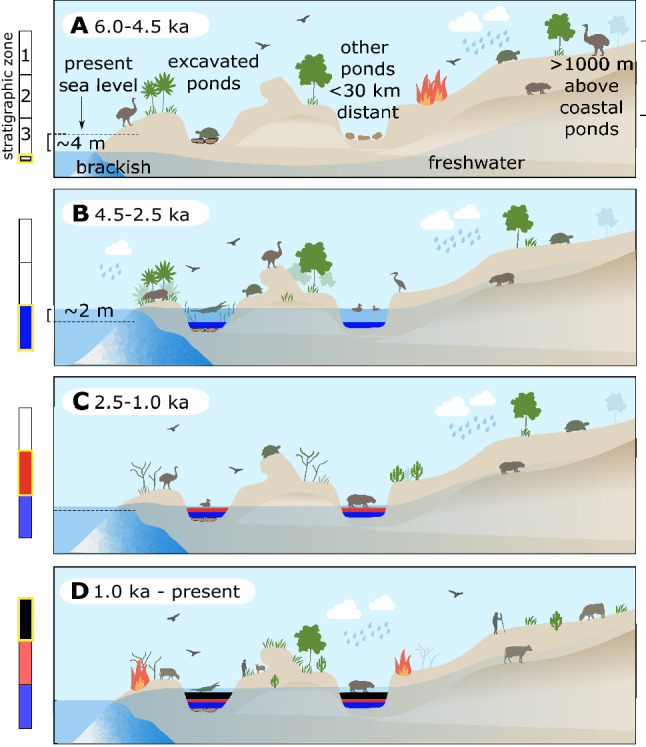
Schematic environmental reconstruction of the study region illustrating changes in relative sea level and climate that contributed sedimentation during relatively wet and dry intervals. Cartoons illustrate changes in vegetation and animal occurrence data from the excavated ponds, other ponds that are < 30 km distant, and sites excavated in the uplands. For example, crocodiles have been ^14^C-dated from the lowland coastal sites during the relatively wet interval ~ 4.5–2.5 ka and were known locally prior to extirpation during the past millennium.

Critically, our data demonstrate the importance of taxon-specific approaches to studying megafaunal extirpation and extinction in the face of changing hunting pressures and resource constraints. For example, an apparent lag between the earliest recorded deposition of giant tortoise bones followed by pygmy hippo bones around Tampolove likely reflects different sensitivities to water scarcity (Figs. 1, 3, and 6C). The excavated sediments record two relatively dry intervals: One ~ 6000–4000 cal BP (when relative sea level was low, Fig. 6A) and the other since ~ 2000 cal BP (during a combination of lowering relative sea level and climatic drying, Fig. 6C). These arid intervals bracket traces of climatic drying in regional records (Fig. 3^28^) and are consistent with a regional pollen record^38^ and other local and regional late Holocene records^24,27,30,36^. Details regarding the interpretation of the excavated Tampolove sediment are given in *Appendix-Discussion-Aridity*.

The relatively limited timespan and late presence of pygmy hippos at sampled low coastal sites such as Tampolove is not a product of sampling bias and reflects (1) their real absence from coastal sites and ranges consequently confined to inland sites until the late Holocene or (2) an early to mid-Holocene occupation of low coastal sites that are currently submerged^26^. In either scenario, the observation remains that pygmy hippos (and crocodiles) were not deposited with giant tortoises in basins around Tampolove before the deposition of lacustrine sediment. Perhaps unsurprisingly, this suggests that pygmy hippos were more sensitive to water scarcity than giant tortoises and elephant birds that occurred in the area before the formation of the ponds. However, though this pattern is clear at the genus level, there are multiple recognized pygmy hippo species on Madagascar^39^, and shifts in species-specific distributions are worth considering in future research (as in other arid regions such as Arabia^40^).

While hippos were apparently absent at Tampolove before sea level approached present levels, there is nevertheless little evidence that hippos were sensitive to later episodes of potential climate drying identified in regional records during 4.5–2.5 ka (Figs. 3 and 6B). Directly ^14^C-dated pygmy hippo remains attest to local persistence during multiple past potential Rodrigues Island “megadroughts” such as that shortly before the resumption of SW Madagascar speleothem formation (3880–3280 cal BP, i.e., continuous occurrence, as in Fig. 1 tortoise). Massive die-offs from severe drought or any other catastrophe are expected to deposit (1) many bones of the same age, and (2) bones of both juveniles and adults. For example, all three of the directly ^14^C-dated hippos from the inland site of Andranosoa have indistinguishable calibrated ^14^C ages (~ 1260–1060 cal BP), which suggests that many could have died during a single event. However, this is not the case at Tampolove, where juvenile bones were scarce and several of the bones spanning the 3880–3280 cal BP arid interval have non-overlapping 95% calibrated ^14^C ages. Bioturbation in zone 3 sediment makes it difficult to distinguish whether this local persistence took place despite local water scarcity or whether climatic drying in the wider SW Indian Ocean region did not end local water availability. The latter is supported by the onset of stalagmite formation at Asafora ~ 3500 cal BP and the relatively low speleothem δ^18^O values during the first part of this record^27^.

The relatively recent deposition of scarce bones, dung fungi, and pollen in zone 2 sediments within the past 2 ka (Figs. 6C and S11–12) may follow from less abundant plant and animal life around the ponds during this later dry interval. Past excavators around Tampolove also noted abundant bones found only under zone 2 sediments, which they described as a “thick layer of white or greyish loam”^41^ and a “whitish chalky layer about six inches or a foot thick”^42^. However, despite the desiccation and apparent disappearance of animals from the excavated ponds, an abundance of giant tortoise (n = 8) and pygmy hippo (n = 11) bone from other ponds and caves in the vicinity of Tampolove have been directly dated to within the past 2 ka. Thus, these animals continued to persist locally despite late Holocene aridity. Note that local persistence of giant tortoises may be particularly cryptic in pond deposits partly because of a shift in where bones were deposited: Between 6 and 3 ka, 9 of 10 directly ^14^C-dated giant tortoises come from the excavated basins, while 9 of 12 directly ^14^C-dated giant tortoises from the following 3 ka come from sheltered overhangs in limestone outcrops (Dataset S6, Fig. S3). This may follow from the fact that tortoises typically die in dry hollows, which no longer existed in coastal basins by 3 ka (Fig. 6A,B). While we do document such changes in patterns of bone deposition during the past 6 ka, there is little evidence that the ranges of giant tortoises and pygmy hippos were contracting away from the vicinity of Tampolove during the arid interval between 2 and 1 ka. Indeed, the last known pygmy hippo from the area likely died around the time of a transition towards relatively wet conditions (Fig. 5).

The marks on the pygmy hippo femur reported here (TAMP-1-2-61) are clear traces of past human activity and similar to chop marks recorded on previously-described pygmy hippo femora from the area (Fig. 4). This increases the plausibility that the previously-excavated femora were marked prior to excavation and not chopped by past excavators who were known to use spades while working in flooded pits with low visibility. Though TAMP-1-2-61 includes clear traces of past human activity, the stratigraphic context makes the perimortem status of the chop marks questionable, and the quality of the chop marks is inconsistent with butchery given that the marks are more likely to extract cortical bone fragments than meat (*Appendix-Discussion-Modified Bone*). The general absence of evidence for hunting in the study area is consistent with the absence of megafaunal bone from coastal shell middens^43^. However, these absences could also easily be explained by preservation bias given we failed to recover cutmarked bone of introduced livestock despite (1) at least a millennium of cattle and goat butchery in the area, and (2) widespread cattle bones in the forests surrounding the excavated ponds (Dataset S1, Fig. S2).

Additionally, megafaunal bones are known from some archaeological sites (e.g., Andranosoa, where three pygmy hippos that died ~ 1260–1060 cal BP were deposited), and cutmarked megafaunal bone is identified elsewhere though it is often the subject of debate^4,44^. Nonetheless, overall, the sediment excavated from Tampolove does not reveal extended coexistence between people and megafauna. Shell fishing may represent an early trace of human presence, for the consistent patterns of damage on the mangrove whelk shells at ANDR (Fig. S13g) are still produced today to extract shellfish meat. However, these shells come from mixed deposits with charcoal that spans the past millennium, and direct dating of estuarine shell is complicated by uncertain marine reservoir corrections. The relatively deep agates (Fig. S13m) are intriguing given that their composition, size, and rounding are inconsistent with local origin. However, they are associated with abundant ratite eggshell and could be merely relatively visible gizzard stones that ratites transported from inland.

Directly ^14^C-dated charcoal fragments and bone records from Tampolove clearly illustrate that increasing fire frequency during the past millennium closely coincided with the local disappearance of megafauna and the arrival of introduced herbivores such as zebu cattle and bushpigs (Figs. 5 and 6D, Table S2). This is consistent with a regional pattern of faunal turnover ~ 1 ka and with the increased influx of charcoal in numerous sedimentary basins in southern Madagascar around the start of the last millennium^4,11,12,15,20,45^. Indeed, given that fires can produce convective columns that transport microcharcoal ~ 10 km, several of the peaks of microcharcoal deposition at Namonte (particularly ~ 1000 and 500 cal BP) may be at least partly explained by fires that burnt the shores of the excavated ponds, leaving occasional burnt bones (e.g., TAMP-1-2-70 at 36 cm depth) and increased deposition of K (Fig. S9). Consistent with recent research, we observe that the charcoal-rich pond sediments deposited during the past millennium include a relatively low diversity of tree pollen^12,38^ and that sediment from recent centuries includes relatively abundant and diverse faecal fungal spores^15^. The positive association between faecal fungal spores and microcharcoal could follow from fires spread to clear habitat of expanding herds of livestock, particularly given that spores of certain fungi that can rely partially on dung (e.g., members of *Coniochaeta*) are notably abundant in soil following fires^46^.

Humans are likely responsible for the abundance of charcoal deposited during both wet and dry times within the past millennium^27^, but we cannot exclude the possibility that less extensive burning shaped the local environment before human arrival. Fire has a long history in the Central Highlands of Madagascar that extends well before the past 10 ka^47^, and the high charcoal concentrations in zone 3 at TAMP and ANKA leave the possibility that there were natural fires in the Southwest during the middle Holocene (Fig. 5). This possibility is consistent with records from Tritrivakely and Ste-Luce^14,48^. However, bioturbation in the ponds around Tampolove contributed to at least some downward movement of macrocharcoal from the past millennium, and the same displacement complicates inference of the undated charcoal record.

Highly-resolved records of past environmental change clarify what may initially seem to be counterintuitive results. For example, animals such as pygmy hippos and giant tortoises responded differently to changing water availability around Tampolove during the past 6 ka, but water scarcity did not coincide with the late Holocene extirpation of particularly drought sensitive taxa (as in Fig. 1). Direct evidence of megafaunal hunting is absent from Tampolove. Though unambiguous traces of human activity here are absent before the last millennium, widespread traces of fire and introduced species starting ~ 1000 cal BP closely coincide with the extirpation of megafauna. Thus, our multidisciplinary data provide strong support for the ‘subsistence shift’ hypothesis of megafaunal extinction on Madagascar (Fig. 2B^8^). To further evaluate what shaped current ecosystems on Madagascar and other islands that lost groups of endemic taxa around the time of human arrival, future research should continue to build occurrence chronologies for other taxa and regions and investigate the stratigraphic context of sites with early traces of human activity.

## Methods

### Excavation sites

During the austral summer of 2019, we surveyed (Dataset S1) and excavated (Datasets S2–S4) the margins of three shallow freshwater ponds in the coastal plains of SW Madagascar: Tampolove [TAMP], Ankatoke [ANKA], and Andranobe [ANDR] (*Appendix-Methods-Survey & Excavation*). These represent a subset of a series of shallow depressions (3–5 m asl) in calcareous crust that define the southern margin of a coastal inlet called the Bay of Assassins (Fig. S1). The crust containing these ponds is bounded by mangrove swamp to the east and both active and ancient dunes to the west (*Appendix-Methods-Site Description*). Each pond is small (< 0.01 km^2^) and has a limited catchment (< 1 km^2^). Coastal ponds in this area have an early history of palaeontological excavations, which recovered rare examples of modified megafaunal bone (*Appendix-Methods–Research History*), yet the last recorded excavations at Tampolove were in 1929^42^. These sites have the current advantage of being within 20 km of a cave (Asafora) that produced a paleoclimate record spanning the past 3.5 ka^27^ and a shallow lake system (Namonte) that produced records of charcoal, pollen, and freshwater diatoms spanning the past 1.2 ka^12^. Plant remains, and subsets of sediment and bones recovered during excavation were exported for analysis. Details regarding all aspects of laboratory analysis are provided in the *Appendix-Methods-Sample Analysis* and *Data Analysis* sections.

### Sediment and Fossils

At the Pennsylvania State University (PSU) Paleoecology Lab, microcharcoal (15–150 µm), macrocharcoal, pollen, and faecal fungal spore concentrations were determined in incremental sediment samples from TAMP (n = 11, Dataset S5). Eleven large (> 1 cm) fragments of charcoal from the three pond excavations were ^14^C dated at the PSU Accelerator Mass Spectrometer (AMS) Lab. Acidified sediment organics (n = 27) were submitted to the Yale Analytical and Stable Isotope Center (YASIC) for elemental and stable carbon and nitrogen isotope (δ^13^C and δ^15^N) analysis. Sediment elemental analysis at 28 depth increments across sites were identified through X-ray fluorescence (XRF) at the PSU Department of Geosciences, and the bulk content of these samples was characterized through drying and combustion techniques at the UC Santa Barbara Department of Earth Sciences. Samples were checked for the presence of diatoms at the lab of M. Velez at the University of Regina and for the presence of foraminifera at the lab of A. Simms at UCSB.

### Bone

Relatively large and complete bone specimens were identified, photographed, and stored in the field headquarters of the Morombe Archaeological Project in Andavadoaka, SW Madagascar. Poorly identified bone fragments of interest (n = 32, deep potentially introduced animal bone or shallow potentially megafaunal bone) were analysed by collagen fingerprinting in the lab of M. Buckley at the University of Manchester. Selected bone specimens recovered from excavation (n = 55) and survey (n = 18) were imaged, sampled, and pretreated for ^14^C analysis at the PSU Human Paleoecology and Isotope Biogeochemistry Lab (Dataset S6). Elemental data were gathered at YASIC or the University of New Mexico’s Center for Stable Isotopes from 43 samples that yielded collagen, and 33 samples of sufficient quality were ^14^C dated at the PSU AMS.

### Data review and analysis

Previously published bone ^14^C data from the study area (n = 41) were compiled, as were island wide bone collagen ^14^C data from extinct pygmy hippos (*Hippopotamus* spp., n = 97) and giant tortoises (*Aldabrachelys* spp., n = 24, Datasets S7 and S8). We used principal components analysis (PCA) to synthesize patterns in sediment XRF data and Bayesian change point analysis (BCPA) to help identify relatively dry and wet intervals in regional paleoclimate records. To help visualize differences in temporal occurrence data at lowland coastal sites versus inland sites, we fitted distributions to ^14^C date count densities. We summed calibrated distributions from charcoal ^14^C data to estimate changes in fire frequency, and we estimated times of local introduced animal arrival and endemic animal extirpation based on sequences of ^14^C data using a Bayesian approach to control for differences in sample size.

## Supplementary Information

Supplementary Information 1.

Supplementary Information 2.

## Data Availability

All data generated or analysed during this study are included in this published article and its supplementary information files.
