# Supplementary material for: Cutmarked bone of drought-tolerant extinct megafauna deposited with traces of fire, human foraging, and introduced animals in SW Madagascar
Source: Sci Rep. 2022 Nov 22;12:18504. doi: 10.1038/s41598-022-22980-w (PMC9681754; doi:10.1038/s41598-022-22980-w)
Supplement: Supplementary file 1 — Supplementary Information 1. [file 41598_2022_22980_MOESM1_ESM.docx]

**Cutmarked Bone of Drought-Tolerant Extinct Megafauna Deposited with Traces of Fire, Human Foraging, and Introduced Animals in SW Madagascar**

Sean W. Hixon^a^, Alejandra I. Domic^b,c^, Kristina G. Douglass^a,d^, Patrick Roberts^a^, Laurie Eccles^c^, Michael Buckley^e^, Sarah Ivory^b^, Sarah Noe^f^, Douglas J. Kennett^f^

^a^ Max Planck Institute for Geoanthropology, Jena, Germany

^b^ Department of Geosciences and the Earth and Environmental Systems Institute, Pennsylvania State University, University Park, PA, USA

^c^ Department of Anthropology, Pennsylvania State University, University Park, PA, USA

^d^ Climate School, Columbia University, New York, NY, USA

^e^ School of Natural Sciences, Manchester Institute of Biotechnology, The University of Manchester, Manchester M1 7DN, UK

^f^ Department of Anthropology, University of California at Santa Barbara, Santa Barbara, CA, USA

**METHODS**

***Site Description***

The coastal plains of SW Madagascar are composed of Quaternary alluvium, which forms dunes and lithified calcareous outcrops that are broken in the east by foothills of Eocene clayey limestone ([Roig et al., 2012](#_ENREF_45)). The climate around the excavation sites (Tampolove [TAMP], Ankatoke [ANKA], and Andranobe [ANDR]) is semi-arid and typically includes <400 mm of annual precipitation, which falls during the austral summer ([November – March, Dewar & Richard, 2007](#_ENREF_14)). The fact that these ponds are near present sea level means that local water availability varies according to changes in both regional precipitation and relative sea level ([Battistini, 1971](#_ENREF_3); [Camoin et al., 2004](#_ENREF_10); [Vallet-Coulomb et al., 2006](#_ENREF_54)).

The excavated ponds are currently shallow enough to permit the growth of emergent plants (plants rooted in lake bottom) throughout, and water levels track variation in rainfall both seasonally and interannually. During the dry season, the relatively verdant ponds are hubs of activity for people and livestock (Fig. S1), and other ponds in the area dry completely. People have constructed spiny bush enclosures around at least parts of each of these ponds to protect the cultivation of crops for human consumption (e.g., sweet potatoes) and emergent plants for fodder and thatching (e.g., reeds such as *Phragmites mauritianus*). The surrounding spiny thicket includes a diversity of endemic baobabs (e.g., *Adansonia grandidieri*), as well as succulents belonging to Didiereaceae (e.g., *Didierea madagascariensis*) and Euphorbiaceae (e.g., *Euphorbia stenoclada*).

TAMP and ANKA are located immediately SE of a coastal village of Vezo fishers known as Tampolove, and ANDR is located immediately SE of a village of Masikoro agropastoralists known as Andalambezo. These villages are less than 10 km west of the Mikea National Park, which contains Lake Namonte and has existed since 2012. The nearest regional population center (~50 km to the NE) is Morombe.


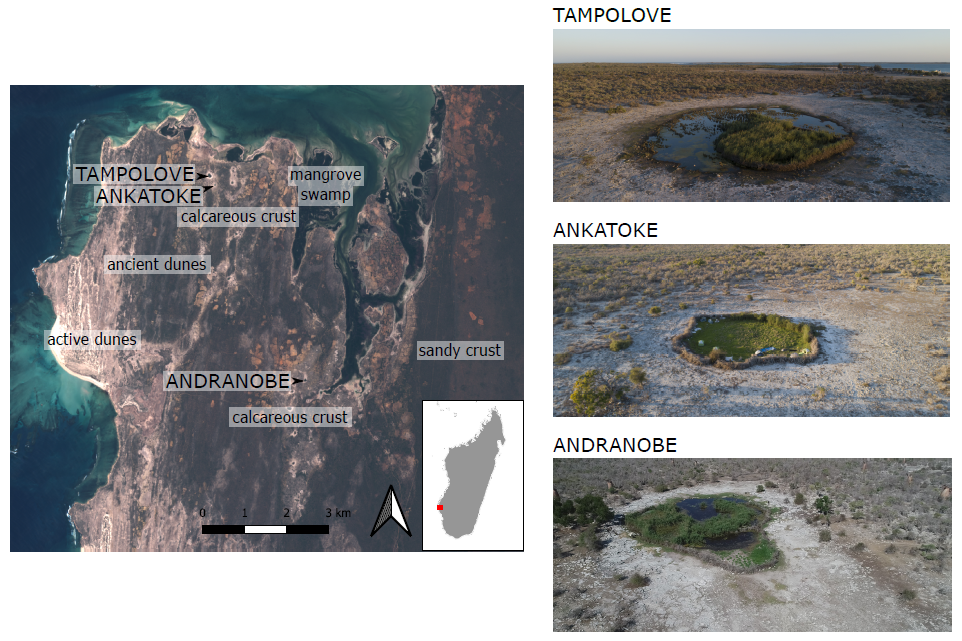


**Figure S1.** *Locations of excavated ponds in the vicinity of Tampolove, SW Madagascar on a Sentinel 2 satellite image. Sandy limestone surrounds each of the basins, but the region includes dunes and mangrove swamps. Each pond is at least partly enclosed for modern cultivation, and only Ankatoke was completely dry during excavation in September, 2019. Map was generated in QGIS (version 3.10.2, www.qgis.org).*

***Research History***

Coastal ponds in the vicinity of Tampolove were the focus of productive paleontological collections of the late 19^th^ and early 20^th^ century that unfortunately lacked stratigraphic context. Shortly before the French colonization replaced the rule of the Sakalava Kingdom over much of W Madagascar, J.T. Last visited “basin-like depressions, now silted up” near the village of “Itampúlu-bé” and recovered abundant remains of elephant bird, pygmy hippo, crocodile, cattle, bushpigs, and other mammals and birds ([Last, 1895, p. 245](#_ENREF_36)). Many of these specimens were sold to the Natural History Museum in London. In the late 1890’s and early 1900’s, Eugène Bastard and Guillaume Grandidier made additional collections from the same taxa in the area for the National Museum of Natural History in Paris, and this included the excavation of coastal ponds ~6 km north of Tampolove and at a site called Lamboharana (near the modern village of Lamboara) ([Grandidier, 1905](#_ENREF_25)). The last expedition to expand museum collections in Europe came through the Vernay-Archbold Expedition of 1929, during which E.I. White excavated a pond in the vicinity of “Itàmpolové” while M. Ramamonjy of the Académie Malgache led additional excavations at Lamboharana/Lamboara ([White, 1930](#_ENREF_60)).

Though Charles Lamberton omitted Itampolove/Tampolove from his map of Malagasy subfossil sites ([Lamberton, 1934](#_ENREF_35)) and created some confusion between the sites of Itampolove and Itampolo ([300 km to the south, see Tattersall, 1987](#_ENREF_50)), bones recovered from Itampolove/Tampolove and the nearby site of Lamboharana/Lamboara significantly shaped our understanding of Malagasy prehistory. Specifically, drilled teeth of the extinct giant aye-aye ([MacPhee & Raholimavo, 1988](#_ENREF_38)) and a couple of modified femora of extinct pygmy hippos directly dated to ~2000 calibrated years before present ([cal BP, MacPhee & Burney, 1991](#_ENREF_39)) from Lamboharana/Lamboara give some of the earliest evidence of interactions between humans and megafauna on the island. However, both sets of modified bone lack stratigraphic context, and the perimortem status of the hippo bone marks has been questioned based on observation that metal blades of excavators can produce clean chopmarks on subfossil bone embedded in stiff sediment ([Anderson et al., 2018](#_ENREF_2)).

The bone assemblages from the vicinity of Tampolove document significant changes in the composition of faunal communities. Specifically, aquatic birds and large, permanent bodies of fresh water are currently scarce. However, the diverse assemblage of subfossil bird bones from Lamboharana/Lamboara (including the extinct Malagasy shelduck (*Alopochen sirabensis*) and Malagasy crowned eagle (*Stephanoaetus mahery*) as well as the extirpated yellow-billed stork (*Mycteria ibis*) and African spoonbill (*Platalea alba*)) reflects a community that likely exploited habitat with relatively abundant surface water during recent millennia ([Goodman & Rakotozafy, 1997](#_ENREF_23)).

***Survey & Excavation***

During July, 2019, ten members of the Morombe Archaeological Project (MAP) completed archaeological survey over an area of ~7 km^2^ in the vicinity of Tampolove. Crew members walked N-S transects while spaced ~10 m apart and systematically recorded and collected surface bones, ceramics, and ratite eggshell (Fig. S2). Survey samples were stored at MAP headquarters in Andavadoaka. Opportunistic survey during August and September 2019 targeted the recovery of megafaunal bone from the surface sediments of nearby coastal rockshelters and the margins of other coastal ponds (Fig. S3).


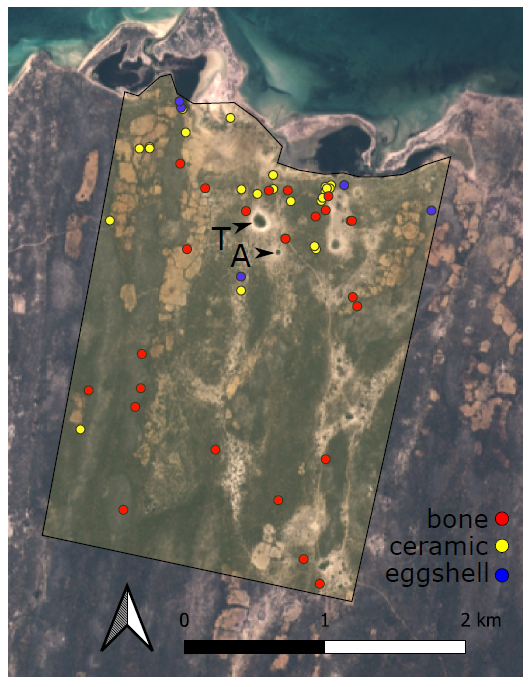


**Figure S2.** *Survey results in in the vicinity of Tampolove (on a Sentinel 2 satellite image), with surveyed area shaded in yellow and color-coded points where different materials were recovered. The Tampolove and Ankatoke excavation sites marked with “T” and “A.” Map was generated in QGIS (version 3.10.2, www.qgis.org).*


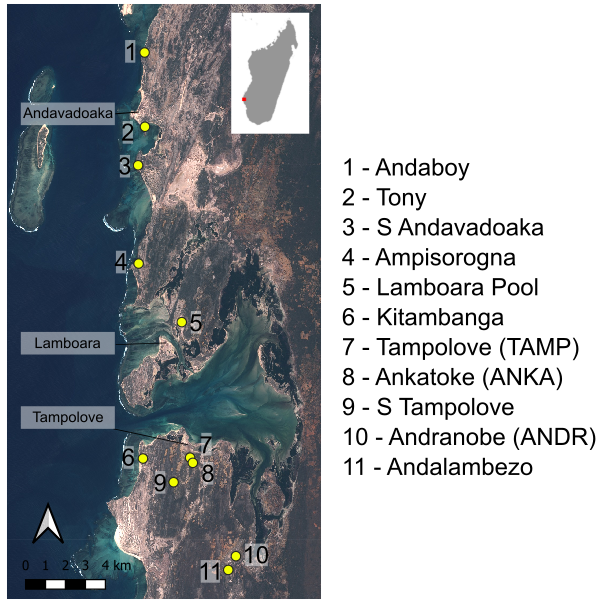


**Figure S3.** *Map illustrating locations of excavated ponds (TAMP, ANKA, ANDR, on a Sentinel 2 satellite image) relative to the modern towns of Andavadoaka, Lamboara, and Tampolove and other sites that produced directly ^14^C dated bone that is discussed in the text (numbered from N to S). Map was generated in QGIS (version 3.10.2, www.qgis.org).*

During August, 2019, we excavated a 1 × 1 m pit at TAMP and 1 × 2 m pits at ANKA and ANDR (Fig. S4). Excavations on the edge of each pond reached ≤120 cm depth below surface (~5 m^3^ of sediment excavated total). We chose to excavate on the muddy margins of each pond to minimize flooding. Groundwater consistently filled pits once we dug below ~50 cm, but this flow was not rapid enough to require routine use of our suction pump. During excavation, we recorded the provenience of all charcoal, wood, bones, ratite eggshell, keratin, and artifacts. We noted the provenience of individual bone fragments when they were scarce. Otherwise, both small bone fragments and any material >2 mm recovered during the wet screening of excavated sediment were assigned to their corresponding layers. We defined layers based primarily on sediment qualities and in some cases on artificial boundaries (Fig. S5-7). Given abundant traces of bioturbation throughout the pond sequences, we used a coarse 10 cm sampling interval during the collection of sediment samples for laboratory analysis. In the field, we noted contacts and fresh sediment Munsell color and used a 14× triplet magnifier to estimate other changes in sediment quality (i.e., grain size, composition, rounding). The bulk of the most common material (megafaunal bone) was labelled, photographed, and stored in plastic bags at MAP headquarters in Andavadoaka. All sediment samples, charcoal, wood, representative samples of artifacts, small animal bones, and bone samples of introduced and endemic animals targeted for chemical analysis were exported to Pennsylvania State University (PSU).


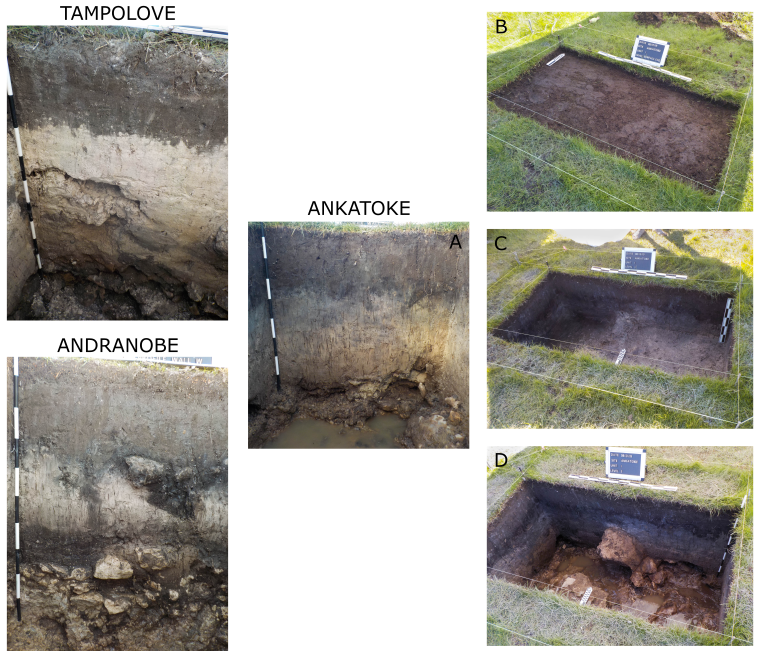


**Figure S4.** *Profiles of Tampolove (W wall), Ankatoke (E wall), and Andranobe (W wall). Errol White likely made extensive excavations at Ankatoke in 1929, and we excavated here a 1 × 2 m trench that passed through surface soil (B), clay (C), and sandy mud around pebbles to boulders (D). Figures S5-7 give digitized field sketches of each profile with full field notes on sediment and organic remains.*


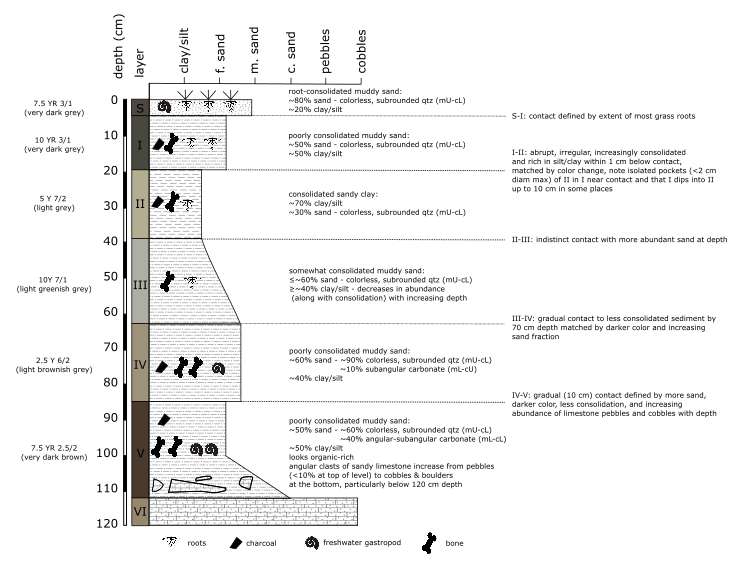


**Figure S5.** *Digitized field notes of Tampolove stratigraphic profile from the SW corner of the pit, with full field notes on sediment and organic remains.*


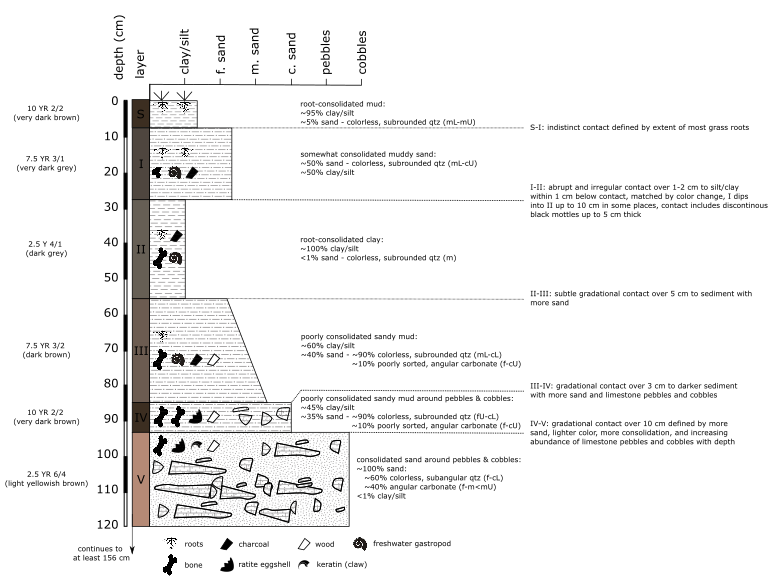


**Figure S6.** *Digitized field notes of Ankatoke stratigraphic profile from the NE corner of the pit, with full field notes on sediment and organic remains.*


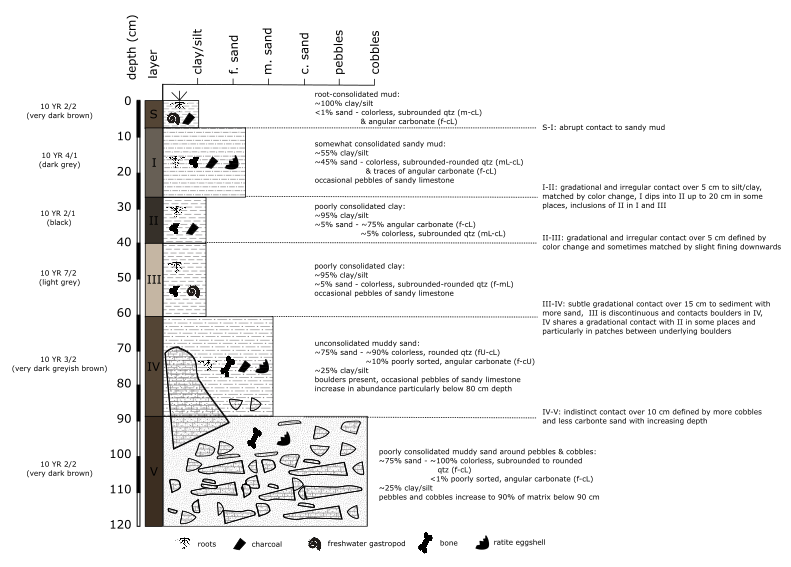


**Figure S7.** *Digitized field notes of Andranobe stratigraphic profile from the SW corner of the pit, with full field notes on sediment and organic remains.*

***Sample Analysis – Sediment & Microfossils***

Sediment XRF: Elemental abundance data from excavated sediment samples (n = 28) were produced using an Olympus DeltaX model x-ray fluorescence (XRF) core scanner and Geotek MSCL 7.9 Multisensor Core Scanner Innoux 1776 at Pennsylvania State University. The trace element composition of sediment is sensitive to a variety of physical and chemical processes that may help explain changes in the diversity of associated plant and animal remains. Specifically, dry conditions and low lake levels may drive relatively more Ca deposition ([Haberzettl et al., 2007](#_ENREF_27); [Kylander et al., 2011](#_ENREF_34)). Deposition of biogenic silica can drive higher Si concentrations ([Agnihotri et al., 2008](#_ENREF_1); [Dickson et al., 2010](#_ENREF_15)), and erosion and fire can deposit mobile elements such as K ([Kylander et al., 2011](#_ENREF_34); [Marion et al., 1991](#_ENREF_40)).

Sediment Bulk Composition & Organics: To quantify the sediment weight percent carbonate at the UCSB Geology Department, we acidified 0.5-0.7 g of <2 mm dried sediment in 20 mL of 1 N HCl overnight at room temperature and differenced the dry masses before and after acidification. To identify weight percent organics, we relied on the approach recommended by Salehi et al. ([2011](#_ENREF_46)) and combusted 2.5 g of <2 mm died sediment at 360°C for 2 h. We also used a multi-step heating method described by Omran ([2016](#_ENREF_42)) to estimate the gypsum content of excavated sediment. Given the low gypsum content of sediment from TAMP and ANDR, we chose not to use this approach with sediment from ANKA. Based on the visible appearance of the excavated sediment, we assume that most of the weight fraction that does not include carbonate, organics, or gypsum includes quartz. The stable carbon isotope (δ^13^C) content of organics in coastal marshes broadly reflect salinity regime, for C4 plants that are relatively tolerant of drought stress and saline environments tend to be enriched in ^13^C (with δ^13^C values of −15‰ to −11‰) relative to C3 plants (with δ^13^C values of −27‰ to −23‰) ([Chmura & Aharon, 1995](#_ENREF_11)). The elemental and stable isotope content of organics from acidified sediment (n = 27) was measured at the Yale Analytical and Stable Isotope Center (YASIC). Secondary standards were used to correct data using two-point normalization, and the mean accuracy of δ^13^C and δ^15^N measurements was 0.1‰. Standard quality assurance data indicate that the mean precision of δ^13^C and δ^15^N measurements across runs was 0.2‰ and 0.1‰, respectively.

Sediment Microcharcoal, Pollen & Spores: Sediment subsamples of 2 cc from TAMP were analyzed for pollen and palynomorph analysis, using standardized pollen techniques, including acetolysis and hydrofluoric acid ([Faegri et al., 1989](#_ENREF_18)). Pollen was too scarce to permit detailed analysis, but fungi spores were relatively common and were identified using palynomorph identification guides ([Demske et al., 2013](#_ENREF_13); [Gauthier & Jouffroy-Bapicot, 2021](#_ENREF_22); [van Geel et al., 2011](#_ENREF_55)). Microcharcoal particles (10-150 µm) were identified on the same pollen slides using a microscope with a magnification of 400×.

Sediment Macrocharcoal: Macrocharcoal in 2 cc sediment subsamples from the three excavated ponds was analyzed following the protocol of Mooney and Radford ([2001](#_ENREF_41)). Additionally, eleven large (1-4 cm) fragments of charcoal from the three pond excavations were pretreated for ^14^C analysis at the PSU Human Paleoecology and Stable Isotope Biogeochemistry (HPSIB) Lab and submitted for analysis at the PSU Accelerator Mass Spectrometer (AMS) Lab. We removed contaminating humates and carbonates from charcoal prior to analysis by pretreating samples with acid/base/acid (ABA) washes that involved 20 min at 60°C with 1N HCl and 1N NaOH followed by repeated rinses in nanopure water ([Kennett et al., 2014](#_ENREF_33)).

***Sample Analysis – Bone***

Bone Protein Fingerprinting: We confirmed the taxonomic assignment of eight animal bones through Zooarchaeology by Mass Spectrometry ([ZooMS, Dataset S3, Buckley et al., 2009](#_ENREF_7)). ZooMS collagen fingerprints were collected from pre-prepared collagen (as below) following Guiry & Buckley ([2018](#_ENREF_26)). In brief, this involved digesting ~1-2 mg collagen with 0.4 μg sequencing grade trypsin (Promega, UK) overnight at 37°C. The digests were then purified and fractionated on solid phase extraction Varian C18 tips (OMIX, UK), into 10% and 50% acetonitrile (ACN) in 0.1% trifluoroacetic acid (TFA), and dried down to completion. Following resuspension in 10 μL 0.1% TFA, 1 μL of each sample was co-crystallised with an equal volume of 10 mg/mL alpha-cyano-hydroxycinnamic acid matrix (in 50% ACN/0.1% TFA), allowed to dry and then analysed by Matrix Assisted Laser Desorption Ionization Time of Flight Mass Spectrometry collecting up to 2,000 laser acquisitions over the mass range *m/z* 700-3,700. The resulting spectra were then compared with reference material from Buckley et al. (2009). Note that, of the 32 samples submitted for protein fingerprinting, 22 (~69%) failed analysis, and one yielded an unidentifiable spectrum. The remaining eight samples included both hippos (n=7) and cattle (n=2), and four of these were directly dated (Fig. S8).

**Figure S8.** *Mass spectra for proteins from four ^14^C-dated animal bones. Ancient specimens are marked with their corresponding ^14^C lab numbers. The 50% fractions for all specimens provided identifiable spectra.*

Bone Imaging & Radiocarbon: We obtained high resolution imagery of key finds (e.g., shallow megafaunal bone fragments and bone cutmarks) using a Keyence VHX-7000 laser scanning digital microscope in the lab of K. Douglass at PSU, and we used a portable Artec Space Spider (Artec 3D, Luxembourg) to create a 3D mesh of the cutmarked pygmy hippo femur before destructive sampling. Bones selected for ^14^C analysis (n = 73 total, 55 from excavation and 18 from survey) were pretreated at the PSU HPSIB and analysed with the PSU AMS. Bones destined for ^14^C analysis were mechanically cleaned, demineralized in 0.5 N hydrochloric acid (HCl) under refrigeration, and gelatinized in 0.01 N HCl at 60°C. Of the 73 bone samples, 29 (~40%) failed protein extraction during demineralization (Table S1). We repeatedly attempted and failed to extract organics from one specimen of particular interest (TAMP-1-2-61) by sampling different parts of the specimen and using relatively gentle demineralization with EDTA ([Tuross et al., 1988](#_ENREF_53)). We purified crude collagen from the remaining 44 samples through ultrafiltration ([Beaumont et al., 2010](#_ENREF_4); [Fernandes et al., 2021](#_ENREF_20)) or XAD resin column chromatography ([Lohse et al., 2014](#_ENREF_37); [Stafford et al., 1988](#_ENREF_47); [Stafford et al., 1991](#_ENREF_48)).

Prior to graphitization and ^14^C analysis, we confirmed bone residue sample quality and protein preservation ([Beaumont et al., 2010](#_ENREF_4); [Kennett et al., 2017](#_ENREF_32); [Van Klinken, 1999](#_ENREF_56)) using both our crude gelatin yields and atomic C:N data (n = 44) gathered from YASIC (n = 41) and the University of New Mexico’s Center for Stable Isotopes (n = 3). Residual contaminants tend to have relatively little N, and we observed relatively high C:N values (>3.5) in 8 bone samples (Table S1). This left 36 specimens for ^14^C analysis, 5 of which were suspected of being duplicates from the same individuals as other specimens. We ultimately chose to ^14^C date 33 specimens that likely come from 31 individuals: A single pygmy hippo from TAMP likely produced a ^14^C-dated right femur (PSUAMS 8666, 1880±20 ^14^C BP) and the fused vertebrae (PSUAMS 8730, 1865±15 ^14^C BP) that we chose to date, and a single giant tortoise from the same site likely produced both a ^14^C-dated plastron fragment (PSUAMS 3955, 3955±25 ^14^C BP) and carapace fragment (PSUAMS 8670, 3925±25 ^14^C BP) that we chose to date.

To expand our subfossil ^14^C dataset, we compiled ^14^C data from all specimens previously collected along a ~30 km stretch of coast around Tampolove (Fig. S3, [Douglass et al., 2019](#_ENREF_16); [Hansford et al., 2021](#_ENREF_28); [Hixon et al., 2021](#_ENREF_29)). This includes data from identified specimens previously gathered in the vicinity of Itampolove/Tampolove (n=8), Lamboharana/Lamboara (n=16), Andavadoaka (n=10), and multiple sites near Andavadoaka (Andaboy, Tony, Ampisorogna, n=7).

**Table S1.** *Summary of bone specimen counts and attrition during pretreatment for ^14^C analysis. Of 73 specimens selected for analysis, 33 were directly dated. When combined with previously published ^14^C data, the total analyzed sample from the vicinity of Tampolove includes data from 74 bone specimens. See main text for explanation regarding the date count from TAMP (*), which includes two duplicate specimens, and note that five previously published ^14^C dates from Tony (Fig. S3) come from ratite eggshell calcite.*

| **Site** | **Sample Sizes** | |  |  |  |
| --- | --- | --- | --- | --- | --- |
|  | Starting | Failed Extraction | C:N>3.5 | Duplicates | ^14^C dated |
| Tampolove (TAMP) | 30 | 14 | 2 | 3 | 13* |
| Ankatoke (ANKA) | 18 | 6 | 2 | 0 | 10 |
| Andranobe (ANDR) | 7 | 1 | 3 | 1 | 2 |
| Multiple - Survey | 18 | 8 | 1 | 1 | 8 |
| Multiple - Prev. Pub. | - | - | - | - | 41 |
| TOTAL | 73 | 29 | 8 | 5 | 74 |

***Data Analysis***

We normalized XRF elemental profiles to Ti, except in the case of Fe, which we normalized to Mn to serve as a ‘palaeo-redox indicator’ given that reduction and release of Mn from sediments precedes that of Fe ([Haberzettl et al., 2007](#_ENREF_27)). We used principal components analysis of normalized data to help synthesize consistent trends in elemental profiles across sites. Unless otherwise noted, all statistical analyses were completed using R version 4.0.3 ([Team, 2013](#_ENREF_51)). We used the R package *bcp* to apply Bayesian change point analysis to previously published paleoclimate records ([Erdman & Emerson, 2007](#_ENREF_17)).

We co-analyzed our local pygmy hippo and giant tortoise ^14^C data with previously published records compiled from island-wide publications (Datasets S7 & S8). The taxonomic resolution of island-wide comparisons between pygmy hippos and giant tortoises is at the genus level, for the multiple species recognized from each may need to be revised ([Goodman et al., 2014](#_ENREF_24)) and are in any case difficult to distinguish based on postcranial bone fragments. Specifically, giant tortoises included *Aldabrachelys grandidieri* and *A. abrupta* ([Burleigh & Arnold, 1986](#_ENREF_8)) and pygmy hippos included multiple classifications involving *Hippopotamus lemerlei*, *H. madagascariensis*, and *H. guldbergi* ([Fovet et al., 2011](#_ENREF_21); [Stuenes, 1989](#_ENREF_49)). Of the 121 previously published records from outside of the Itampolove/Tampolove study area, 15 have questionable reliability due to C:N>3.6 or limited ^14^C pretreatment or quality control information. However, given that these entries of the compiled dataset are used for comparisons at broad (millennial) timescales, potentially unreliable entries were not excluded from analysis.

All ^14^C dates were calibrated in OxCal 4.4 using the Southern Hemisphere calibration curve SHCal20 ([Hogg et al., 2020](#_ENREF_30)) or the post-bomb atmospheric SH3 ([Hua et al., 2013](#_ENREF_31)) for three specimens with >^14^C modern ages. To help visualize the calibrated distributions of charcoal ^14^C data from the study sites, we used the package *rcarbon* to sum these distributions ([Bevan & Crema, 2020](#_ENREF_5)). To characterize and compare distributions of mean calibrated ages from giant tortoises and pygmy hippos at inland and low coastal sites (Fig. 1), we fit Gamma(α,β) distributions to each positively skewed data set using maximum likelihood estimations with *fitdistr* function ([Ripley et al., 2013](#_ENREF_44)). To create confidence intervals for local herbivore arrival and extirpation based on ^14^C dated occurrence datasets, we used a Bayesian approach ([Buck & Bard, 2007](#_ENREF_6)) in OxCal 4.4 ([Ramsey, 2009](#_ENREF_43)). This followed the same assumptions as specified by Hixon et al. ([2021](#_ENREF_29)), and was informed by reliably dated remains within the past ~4,000 years from hippos (n = 26), giant tortoises (n = 18), and zebu (n = 9).

**RESULTS**

***Survey***

Surface survey in the vicinity of Tampolove recovered a somewhat even spread of surface bone, while ceramics and ratite eggshell fragments were concentrated relatively close to the shore (Fig. S2, Dataset S1). Zebu cattle were most commonly represented in the surface bone scatters, but small tortoises, small reptiles, and ovicaprids or bushpigs were also collected. Only one bone of extinct megafauna was recovered from the surface within the Tampolove study area, and it was a giant tortoise carapace fragment found under an inland limestone ledge near the SW corner of the survey area in Fig. S2. Local informants from Tampolove told stories of zebu that occasionally became entrapped in the thick sediment of nearby coastal ponds and mentioned that large bones not belonging to zebu occasionally surface on the margins of these ponds during cultivation.

Opportunistic survey of other coastal rock shelters near Kitambanga (Fig. S3) and Andavadoaka recovered three additional specimens of giant tortoise carapace. Similar surface survey of coastal ponds near Andalambezo and Lamboara recovered bone fragments of pygmy hippo, giant tortoise, crocodile, and zebu cattle. A local guide led us to the pond near Andalambezo where he had unearthed pygmy hippo and giant tortoise bone fragments while cultivating sweet potatoes. Bone fragments from Lamboara came from the surface of eroding heaps of sediment immediately adjacent to hollowed depressions in the limestone.

***Excavation***

Fossiliferous sandy marine limestone defines the base of each profile (Fig. S5-7 & S9, TAMP VI, ANKA V, ANDR V). This limestone crops out on the margin of each pond, and the fossils include primarily marine gastropods (e.g., *Gibberulus albus* & *Rhinoclavis vertagus*) but also ratite eggshell at ANDR. This sandy limestone occurs as cobbles and boulders that surround few to no organic remains at the base of each excavated trench. The stratigraphy of the overlying soft sediment includes shared patterns among all three ponds. This is most visibly defined by a layer of clay (zone 2 in Fig. S9), which separates the surface soil formation (zone 1) from the underlying fossiliferous muddy sand and bedrock (zone 3).

*Zone 3*. Intermediate organic content and relatively high Fe/Mn values distinguish zone 3 (TAMP IV-V, ANKA III-IV, ANDR IV) from the overlying sediment (Dataset S2). The organic δ^13^C values are consistently low (n = 10, x̄ = −24.5‰, SD = 0.9‰, Fig. S10), and no forams or diatoms are present in this or the overlying sediment. Trace quantities of gypsum are present at TAMP and ANDR in zones 1-3, yet XRF detected sulfur only at TAMP and only in zone 3. By dry weight, sediment in this zone is dominated by carbonate (~72-89%). The carbonate component is poorly sorted and angular, with fragments of planorbid snail shell and some clasts of limestone. Colorless, rounded, medium-coarse quartz sand (similar to that in dunes to the west) accounts for most of the remaining sediment weight (<25%). TAMP sediment includes nematode eggs, hyphae, and spores belonging to multiple taxa of dung fungi (e.g., *Ascodesmis* and *Podospora*, Fig. S11). Spores of fungi associated with plants are also present, and these include a mycorrhizal fungus belonging to *Glomus*. Pollen from grass (Poaceae) and a diversity of trees, shrubs, and aquatic plants are present (Fig. S12), as are seeds of sedges (Cyperaceae).

*Zone 2*. A gradational contact that spans 5-10 cm separates the sediment of zone 3 from the light grey consolidated clay of zone 2 (TAMP II-III, ANKA II, ANDR II-III). The organic content of zone 2 is the lowest of the three recognized zones (1-5 wt. %), and acidified sediment samples have relatively little N (0.6-2.6 wt. %). The few organics that are present have δ^13^C values that increase upwards through this zone by up to 4‰ (in the case of TAMP). Zone 2 sediment has high Ca/Ti values despite containing relatively less carbonate than zone 3 (~44-84%). Fragments of planorbid snail shells are relatively scarce in zone 2, and <1 mm calcite crystals are relatively abundant. At each site, zone 2 includes local maxima in the deposition of quartz (as much as 51 wt. % at ANKA, 50-60 cm depth), which was difficult to recognize in the field and not included in profile sketches. TAMP zone 2 sediment includes breaks in the deposition of multiple microfossils, including dung fungi (e.g., *Ascodesmis* & *Podospora*), nematode eggs, tree pollen (e.g., *Euphorbia* & *Fabaceae*), grass pollen, and pollen from multiple emergent plant taxa (*Carex* and *Typha*). However, Cyperaceae pollen is present throughout zone 2 sediment, and both generalist fungi (*Nigrospora*) and plant decomposing fungi (*Anthostomella*) are present through most of the zone.

*Zone 1*. An abrupt and irregular contact separates the surface sediment from the underlying clay of zone 2, yet clay inclusions from zone 2 (≤5 cm diameter) occur in zone 1 immediately above the contact. Intermediate Ca/Ti values, coupled with relatively high Si/Ti and K/Ti values, characterize the surface sediment and soil formation of zone 1 (TAMP S-I, ANKA S-I, ANDR S-I). Zone 1 sediment includes the highest organic content of the three recognized zones, and these organics are enriched in ^13^C (n = 7, x̄ = −20.5‰, SD = 2.2‰) relative to the underlying organics (n = 19, x̄ = −24.2‰, SD = 1.4‰, t(24) = 5.0, p < 0.001). Quartz sand is increasingly abundant toward the top of zone 1 at TAMP and ANKA (up to 68 wt. % at TAMP), but this is indistinct in the muddy root mat that forms the surface of each profile. TAMP zone 1 sediment includes nematode eggs and spores from a relatively great diversity of fungi (including the only observed *Sporormiella* spores). The concentration of pollen in zone 1 is relatively low. With the exception of *Ambrosia* (with pollen recognized only in zone 1 sediment), terrestrial and aquatic plant pollen in zone 1 sediment comes from a subset of the taxa recognized in zone 3 sediment (6 of 14).

Details regarding faunal remains are given in the main text, and illustrative summaries of dated material from TAMP, ANKA, and ANDR (Figs. S13-S16) are given below, as is a table that summarizes the sequence of directly ^14^C dated charcoal from these sites (Table S2).


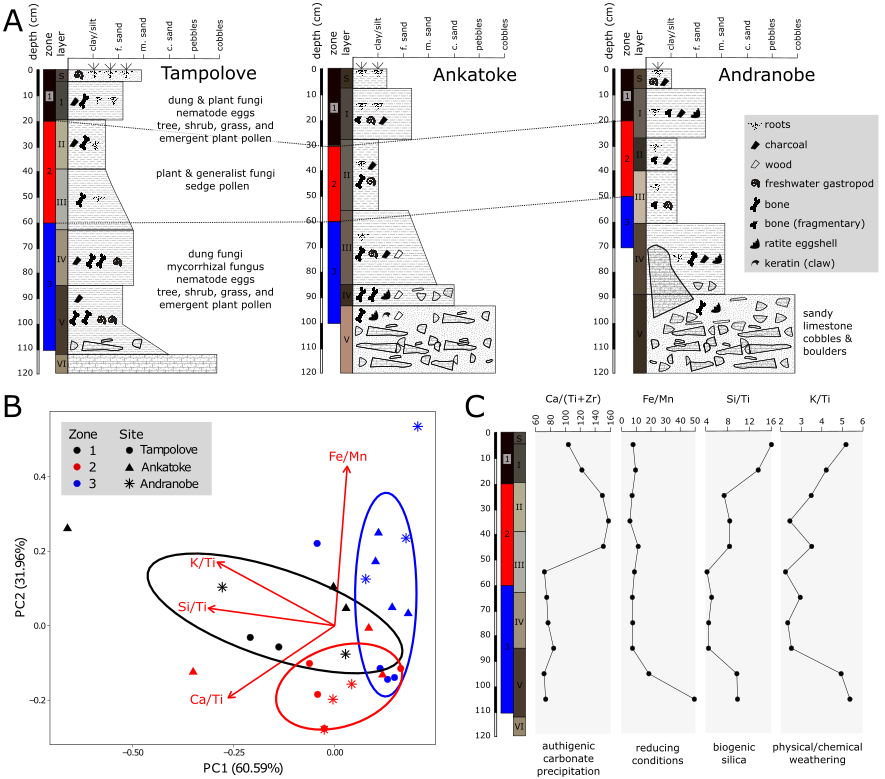


**Figure S9.** *Lithology and trace element content of excavated pond sediments, including sketches of stratigraphic profiles based on field notes (A, Figs. S5-S7), principal components analysis of sediment XRF data with ellipses that outline 68% of the data from each group (B), and XRF profiles from Tampolove for representative examples (C). Full displays of fossil and microfossil data exist in Figs. S9-13. Each sequence in (A) includes a consolidated unit with grey clay (zone 2) that separates underlying megafaunal bone beds (including zone 3) from overlying soils (zone 1). These zones are similar across sites and are geochemically distinct (B).*


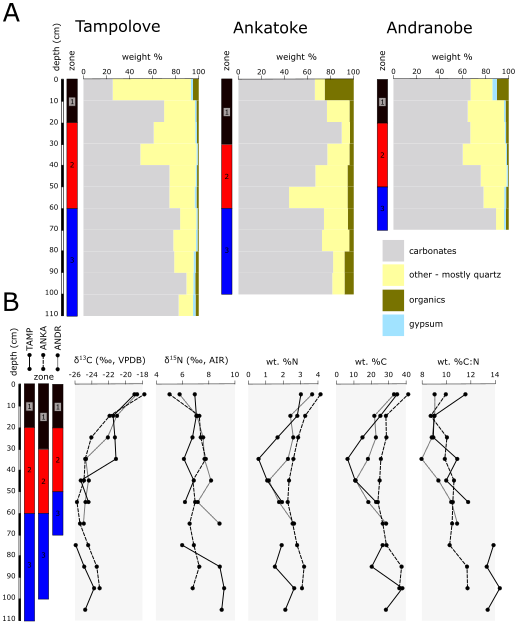


**Fig. S10.** *Summaries of changes in sediment composition (A) and sediment organics attributes (B) from the three excavated sites. The three color-coded stratigraphic zones match those in Fig. S9. Note that gypsum content was estimated only at TAMP and ANDR, and that the “other” category in (A) includes primarily quartz and is calculated after direct measurements of carbonate, organic, and gypsum content.*


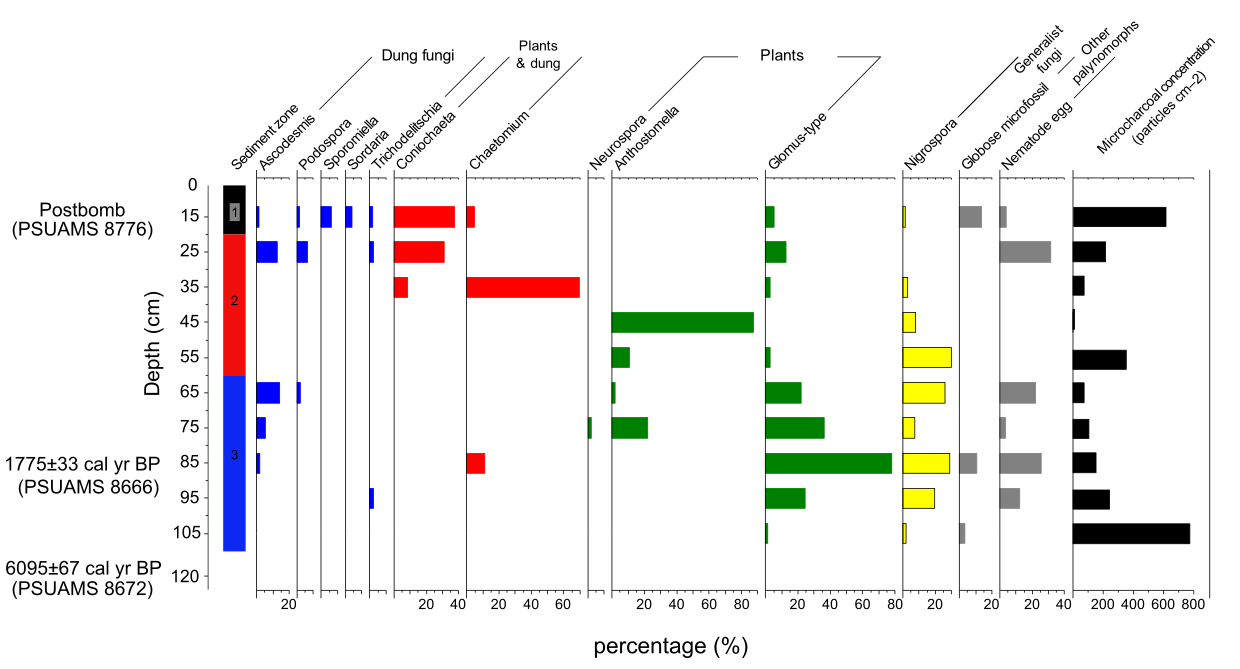


**Fig. S11.** *Fungi, other palynomorphs, and microcharcoal identified and counted from TAMP sediment samples versus depth. Fungi are grouped and colored based on their associations with dung, plants, and other materials (Dataset S9). Stratigraphic zones are marked to ease comparison with Fig. S9. Three ^14^C dates (including the youngest and oldest from zone 3 sediment) are shown here against depth, and a complete illustration of ^14^C data from TAMP is in Fig. S14.*


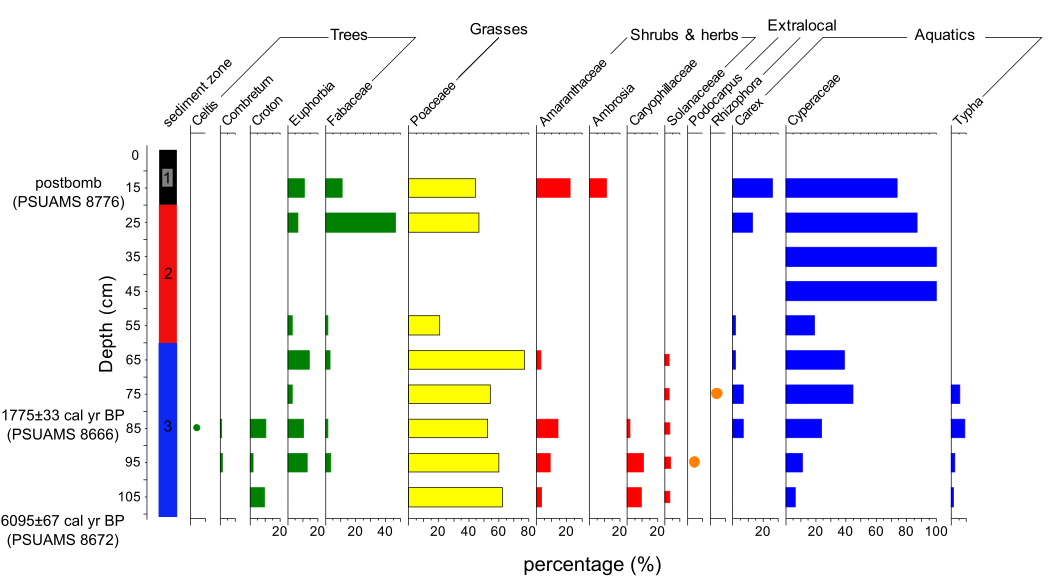


**Fig. S12.** *Pollen identified and counted from TAMP sediment samples versus depth. As in Fig. S11, stratigraphic zones are marked to ease comparisons, and three ^14^C dates are shown here against depth as examples.*


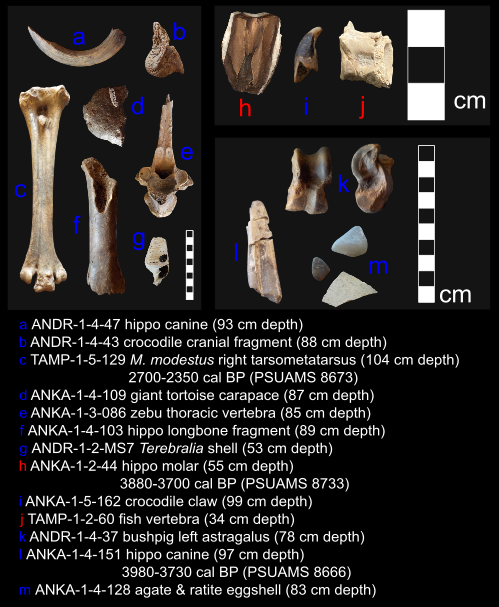


**Figure S13.** *Examples of material recovered from Tampolove (TAMP), Ankatoke (ANKA), and Andranobe (ANDR) and discussed in the main text. These come from stratigraphic zones 2 and 3 (red and blue, respectively), with corresponding 2σ calibrated age ranges for three directly specimens and depths below surface given in parentheses.*


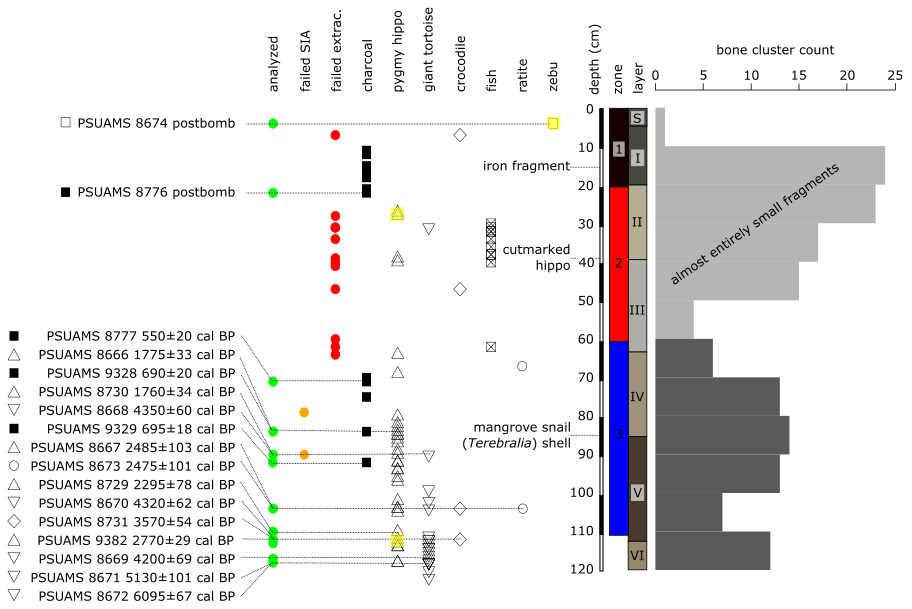


**Figure S14.** *Faunal and charcoal occurrence and ^14^C data from Tampolove, with colored layers and zones from Fig. S9 for reference. Green circles mark depths of ^14^C-dated plant and animal remains, orange circles mark depths of bones that yielded collagen with signs of contamination, and red circles mark depths of bones that failed collagen extraction. Depths of only confidently-identified bones are shown, and points are shaped according to taxon. Specimens identified through protein fingerprinting are highlighted in yellow, and six specimens that failed identification through protein fingerprinting come from layers I-III. Associated ^14^C data are given at left with ages (µ±σ) calibrated using SHCal20. Note that two directly dated pygmy hippo bones have indistinguishable ^14^C dates and may come from the same individual, and the same is true for two directly dated giant tortoise bones. The histogram at right gives counts of depth-recorded bone clusters binned at 10 cm increments. Note that very few of the small bone fragments in the upper section of the sequence can be confidently identified and that the cutmarked hippo referenced in this figure corresponds to TAMP-1-2-61 in Fig. 4.*


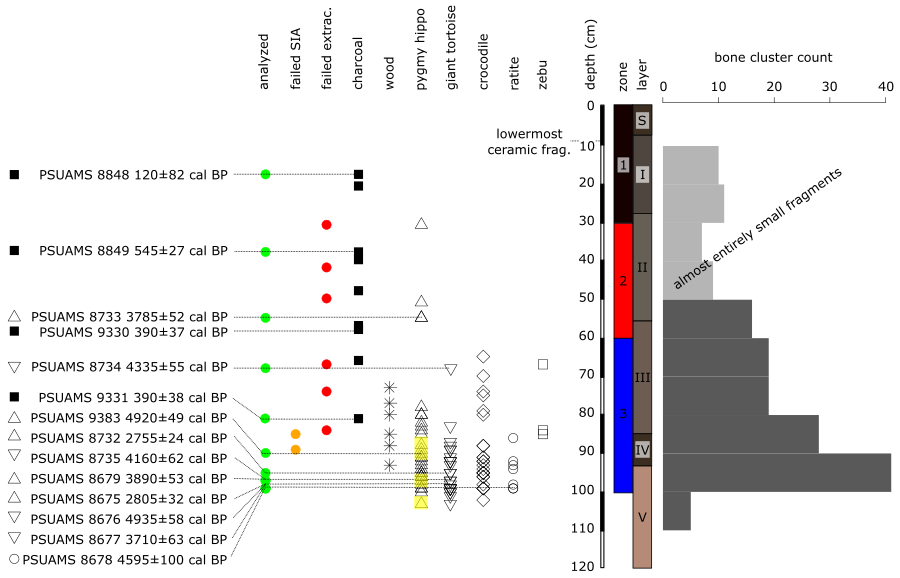


**Figure S15.** *Faunal and charcoal occurrence and ^14^C data from Ankatoke, with colored layers and zones from Fig. S9 for reference. Green circles mark depths of ^14^C-dated plant and animal remains, orange circles mark depths of bones that yielded collagen with signs of contamination, and red circles mark depths of bones that failed collagen extraction. Depths of only confidently-identified bones are shown, and points are shaped according to taxon. Specimens identified through protein fingerprinting are highlighted in yellow. Associated ^14^C data are given at left with ages (µ±σ) calibrated using SHCal20. The histogram at right gives counts of depth-recorded bone clusters binned at 10 cm increments. Note that very few of the small bone fragments in the upper section of the sequence can be confidently identified.*


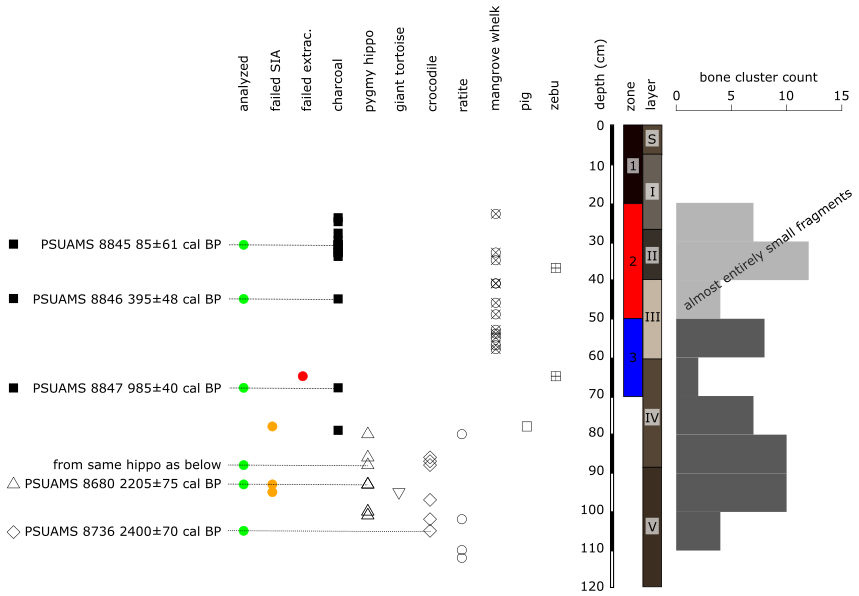


**Figure S16.** *Faunal and charcoal occurrence and ^14^C data from Andranobe, with colored layers and zones from Fig. S9 for reference. Green circles mark depths of ^14^C-dated plant and animal remains, orange circles mark depths of bones that yielded collagen with signs of contamination, and red circles mark depths of bones that failed collagen extraction. Depths of only confidently-identified bones are shown, and points are shaped according to taxon. Associated ^14^C data are given at left with ages (µ±σ) calibrated using SHCal20. The histogram at the right gives counts of depth-recorded bone clusters binned at 10 cm increments. Note that very few of the small bone fragments in the upper section of the sequence can be confidently identified.*

**Table S2.** *Sequence of charcoal ^14^C data from the excavated sediment of TAMP, ANKA, and ANDR (Figs. 5 & S14-S16). There are multiple examples of similarly aged specimens that come from different sites and depths.*

| site (level) | Depth (cm) | Lab code | conventional | 2 sigma cal BP |
| --- | --- | --- | --- | --- |
| TAMP | 22 | PSUAMS 8776 | −2575±35 | −12- −26 |
| ANDR I | 31 | PSUAMS 8845 | 110±20 | 250-5 |
| ANKA I | 18 | PSUAMS 8848 | 155±20 | 265-0 |
| ANKA II | 57 | PSUAMS 9330 | 360±15 | 450-315 |
| ANKA III | 81 | PSUAMS 9331 | 365±15 | 450-315 |
| ANDR II | 45 | PSUAMS 8846 | 380±20 | 490-320 |
| ANKA II | 38 | PSUAMS 8849 | 575±30 | 625-505 |
| TAMP IV | 71 | PSUAMS 8777 | 590±15 | 625-525 |
| TAMP IV | 84 | PSUAMS 9328 | 795±20 | 725-660 |
| TAMP IV | 92 | PSUAMS 9329 | 805±20 | 725-670 |
| ANDR IV | 68 | PSUAMS 8847 | 1120±20 | 1050-930 |

**DISCUSSION**

***Aridity***

Traces of two dry intervals are present in the excavated sediment. The first is most clearly visible at the base of TAMP (~6,000-4,000 cal BP), where giant tortoise carapace fragments spanning multiple millennia were deposited together and with no separation by lacustrine sediments (Fig. 6 & S14). Relative sea level was low compared to present (−2 to −3 m), and coastal water tables were likely also relatively low given that pollen from Ambolisatra/Andolonomby documents the contraction of humid woodland (including *Uapaca* cf. *bojeri*) and expansion of dry-adapted spiny bush (Didiereaceae) by ~5500 cal BP ([Virah-Sawmy et al., 2016](#_ENREF_58)). A hiatus in speleothem formation in SW Madagascar during the early to mid Holocene also suggests that this interval was relatively dry ([Burns et al., 2022](#_ENREF_9)). The second dry interval at Tampolove is reflected in the sediment of zone 2 (poorly constrained in time, but <2,000 cal BP), which lacks the traces of a waterlogged reducing environment that are present in the underlying zone 3 sediment (e.g., sediment with high Fe/Mn, well-preserved organic remains, emergent plant seeds and a diversity of emergent plant pollen, waterfowl bones, spores from mycorrhizal fungi, and abundant planorbid snail shells). The high Ca/Ti values and quartz content of zone 2 reflect authigenic carbonate precipitation (visible as calcite crystals) and relatively large allochthonous inputs, both of which are consistent with low water levels and aquatic primary production. Zone 2 sediment deposition could be explained by a combination of lowering relative sea level and climatic drying that is documented in both local and regional records ([Camoin et al., 2004](#_ENREF_10); [Faina et al., 2021](#_ENREF_19); [Vallet-Coulomb et al., 2006](#_ENREF_54); [Wang et al., 2019](#_ENREF_59)). While these ponds might have been dry or very shallow, the relatively low sediment δ^13^C values, low abundance of sulfur, and absence of marine microfossils suggest that these ponds remained freshwater ponds. The increasing δ^13^C values up zones 1 and 2 are likely explained by small rootlets of grasses that currently grow on the margins of these ponds.

***Modified Bone***

Its context makes the perimortem status of the TAMP-1-2-61 chopmarks questionable, and the quality of the chopmarks is inconsistent with butchery. The cutmarked femur is stratigraphically removed from the abundance of relatively large megafaunal bone in zone 3 by ~40 cm, and it is >20 cm above 3 large fragments of charcoal that date to <700 cal BP. While it is possible that the TAMP-1-2-61 femur comes from a pygmy hippo that died within the past 700 years (the bone failed direct ^14^C analysis), this possibility is unlikely given that none of the pygmy hippos from the island that have been directly dated thus far (n = 118) postdate 900 cal BP. The possibility that TAMP-1-2-61 is older than the underlying charcoal is supported by the example of the directly ^14^C-dated pygmy hippo molar fragment from ANKA zone 2 sediment that is >3 ka older than an associated charcoal fragment. The fact that TAMP-1-2-61 is by far the largest bone fragment from zone 2 sediment also makes it an outlier and suggests that the bioturbation that brought it to a shallow depth may be different than the bioturbation responsible for mixing relatively small fragments of charcoal, teeth, and long bones. Indeed, unlike ANKA and ANDR, the upper section of TAMP zone 2 (~25-45 cm depth) has relatively constant sediment Ca/Ti and organics δ^13^C values that are distinct from underlying values (former are consistently ~3‰ greater, Fig. S10), which is consistent with an event of massive sediment deposition. The likely antiquity of TAMP-1-2-61 and its shallow stratigraphic context leave the possibility that the bone was modified following the death of the animal.

The smooth surfaces of the chop marks do suggest that the bone was modified before the loss of bone protein left it brittle and chalky. However, the exceptional preservation of organics in zone 3 highlights the fact that the loss of bone structural proteins can lag the death of the given animal by millennia. Consequently, the “fresh” status of the bone at the time of modification cannot sufficiently support the conclusion that the marks were perimortem. This is particularly true given that people still regularly unearth megafaunal bone while cultivating the margins of ponds around Tampolove (Appendix Results).

Additionally, perimortem cutmarks for meat extraction are expected to be deep, narrow, nearly perpendicular to the bone shaft, and near joints. However, as seen in Figure 4, the marks on TAMP-1-2-61 form broad planes that pass through the entire shaft and in one noticeable case run along the bone shaft. This damage pattern is more likely to extract thick chips of cortical bone than meat. Past bone tool use is undescribed on Madagascar but known from the archaeological records of other islands. For example, on New Zealand, the Maori used moa bone to create fish hooks ([Teviotdale, 1929](#_ENREF_52)) and whale bone to manufacture a variety of artifacts ([Cunliffe & Brooks, 2016](#_ENREF_12)). Given the association between TAMP-1-2-61 and the cutmarked fishbone, it is plausible that a person used TAMP-1-2-61 to create tools associated with fishing. However, there are multiple possibilities, and similarly notched bone of zebu was also previously recovered from the inland archaeological site of Rezoky ([Vérin & Battistini, 1971](#_ENREF_57)), which has bone deposits that span ~730-500 cal BP ([Hixon et al., 2021](#_ENREF_29)).

References

Agnihotri, R., Altabet, M. A., Herbert, T. D., & Tierney, J. E. (2008). Subdecadally resolved paleoceanography of the Peru margin during the last two millennia. *Geochemistry, Geophysics, Geosystems, 9*(5).

Anderson, A., Clark, G., Haberle, S., Higham, T., Nowak-Kemp, M., Prendergast, A., . . . Virah-Sawmy, M. (2018). New evidence of megafaunal bone damage indicates late colonization of Madagascar. *PloS one, 13*(10), e0204368.

Battistini, R. (1971). Chronologie du Quaternaire littoral de Madagascar. *Bull. Liaison Assoc. S~ n~ gal. Quatern. Ouest Aft, 31*, 23-30.

Beaumont, W., Beverly, R., Southon, J., & Taylor, R. (2010). Bone preparation at the KCCAMS laboratory. *Nuclear Instruments and Methods in Physics Research Section B: Beam Interactions with Materials and Atoms, 268*(7), 906-909.

Bevan, A., & Crema, E. (2020). rcarbon v1. 4.1: Calibration and Analysis of Radiocarbon Dates. *rcarbon v1. 4.1: Calibration and Analysis of Radiocarbon Dates*.

Buck, C. E., & Bard, E. (2007). A calendar chronology for Pleistocene mammoth and horse extinction in North America based on Bayesian radiocarbon calibration. *Quaternary Science Reviews, 26*(17-18), 2031-2035.

Buckley, M., Collins, M., Thomas‐Oates, J., & Wilson, J. C. (2009). Species identification by analysis of bone collagen using matrix‐assisted laser desorption/ionisation time‐of‐flight mass spectrometry. *Rapid Communications in Mass Spectrometry: An International Journal Devoted to the Rapid Dissemination of Up‐to‐the‐Minute Research in Mass Spectrometry, 23*(23), 3843-3854.

Burleigh, R., & Arnold, E. (1986). Age and dietary differences of recently extinct Indian Ocean tortoises (Geochelone s. lat.) revealed by carbon isotope analysis. *Proc. R. Soc. Lond. B, 227*(1246), 137-144.

Burns, S. J., McGee, D., Scroxton, N., Kinsley, C. W., Godfrey, L. R., Faina, P., & Ranivoharimanana, L. (2022). Southern Hemisphere controls on ITCZ variability in southwest Madagascar over the past 117,000 years. *Quaternary Science Reviews, 276*, 107317.

Camoin, G., Montaggioni, L., & Braithwaite, C. (2004). Late glacial to post glacial sea levels in the Western Indian Ocean. *Marine Geology, 206*(1), 119-146.

Chmura, G., & Aharon, P. (1995). Stable carbon isotope signatures of sedimentary carbon in coastal wetlands as indicators of salinity regime. *Journal of Coastal Research*, 124-135.

Cunliffe, E., & Brooks, E. (2016). Prehistoric whale bone technology in southern New Zealand. *International Journal of Osteoarchaeology, 26*(3), 384-396.

Demske, D., Tarasov, P. E., & Nakagawa, T. (2013). Atlas of pollen, spores and further non-pollen palynomorphs recorded in the glacial-interglacial late Quaternary sediments of Lake Suigetsu, central Japan. *Quaternary International, 290*, 164-238.

Dewar, R. E., & Richard, A. F. (2007). Evolution in the hypervariable environment of Madagascar. *Proceedings of the National Academy of Sciences, 104*(34), 13723-13727.

Dickson, A. J., Leng, M. J., Maslin, M. A., & Röhl, U. (2010). Oceanic, atmospheric and ice-sheet forcing of South East Atlantic Ocean productivity and South African monsoon intensity during MIS-12 to 10. *Quaternary Science Reviews, 29*(27-28), 3936-3947.

Douglass, K., Hixon, S., Wright, H. T., Godfrey, L. R., Crowley, B. E., Manjakahery, B., . . . Radimilahy, C. (2019). A critical review of radiocarbon dates clarifies the human settlement of Madagascar. *Quaternary Science Reviews, 221*, 105878.

Erdman, C., & Emerson, J. W. (2007). bcp: an R package for performing a Bayesian analysis of change point problems. *Journal of statistical software, 23*(1), 1-13.

Faegri, K., Kaland, P. E., & Krzywinski, K. (1989). *Textbook of pollen analysis*: John Wiley & Sons Ltd.

Faina, P., Burns, S. J., Godfrey, L. R., Crowley, B. E., Scroxton, N., McGee, D., . . . Ranivoharimanana, L. (2021). Comparing the paleoclimates of northwestern and southwestern Madagascar during the late Holocene: implications for the role of climate in megafaunal extinction. *Malagasy Nature, 15*, 108-127.

Fernandes, D. M., Sirak, K. A., Ringbauer, H., Sedig, J., Rohland, N., Cheronet, O., . . . Culleton, B. J. (2021). A genetic history of the pre-contact Caribbean. *Nature, 590*(7844), 103-110.

Fovet, W., Faure, M., & Guérin, C. (2011). Hippopotamus guldbergi n. sp.: révision du statut d'Hippopotamus madagascariensis Guldberg, 1883, après plus d'un siècle de malentendus et de confusions taxonomiques. *Zoosystema, 33*(1), 61-82.

Gauthier, E., & Jouffroy-Bapicot, I. (2021). Detecting human impacts: non-pollen palynomorphs as proxies for human impact on the environment. *Geological Society, London, Special Publications, 511*(1), 233-244.

Goodman, S., & Rakotozafy, L. (1997). Subfossil birds from coastal sites in western and southwestern Madagascar: A paleoenvironmental reconstruction. In S. Goodman & B. Patterson (Eds.), *Natural Change and Human Impact in Madagascar* (pp. 257-279): Smithsonian Institution Press,.

Goodman, S. M., Jungers, W. L., & Simeonovski, V. (2014). *Extinct Madagascar: Picturing the Island's Past*: University of Chicago Press.

Grandidier, G. (1905). Les animaux disparus de Madagascar. Gisements, époques et causes de leur disparition. *Revue de Madagascar, 7*, 111-128.

Guiry, E., & Buckley, M. (2018). Urban rats have less variable, higher protein diets. *Proceedings of the Royal Society B, 285*(1889), 20181441.

Haberzettl, T., Corbella, H., Fey, M., Janssen, S., Lücke, A., Mayr, C., . . . Wille, M. (2007). Lateglacial and Holocene wet—dry cycles in southern Patagonia: chronology, sedimentology and geochemistry of a lacustrine record from Laguna Potrok Aike, Argentina. *The Holocene, 17*(3), 297-310.

Hansford, J. P., Lister, A. M., Weston, E. M., & Turvey, S. T. (2021). Simultaneous extinction of Madagascar's megaherbivores correlates with late Holocene human-caused landscape transformation. *Quaternary Science Reviews, 263*, 106996.

Hixon, S. W., Douglass, K. G., Crowley, B. E., Rakotozafy, L., Clark, G., Anderson, A., . . . Kennett, D. (2021). Late Holocene spread of pastoralism coincides with endemic megafaunal extinction on Madagascar. *Proc. R. Soc. Lond. B, 288*(1955), 20211204.

Hogg, A. G., Heaton, T. J., Hua, Q., Palmer, J. G., Turney, C. S., Southon, J., . . . Ramsey, C. B. (2020). SHCal20 Southern Hemisphere calibration, 0–55,000 years cal BP. *Radiocarbon*, 1-20.

Hua, Q., Barbetti, M., & Rakowski, A. Z. (2013). Atmospheric radiocarbon for the period 1950–2010. *Radiocarbon, 55*(4), 2059-2072.

Kennett, D., Plog, S., George, R., Culleton, B., Watson, A., Skoglund, P., . . . Perry, G. (2017). Archaeogenomic evidence reveals prehistoric matrilineal dynasty. *Nature Communications*.

Kennett, D. J., Culleton, B. J., Dexter, J., Mensing, S. A., & Thomas, D. H. (2014). High-precision AMS 14 C chronology for Gatecliff Shelter, Nevada. *Journal of Archaeological Science, 52*, 621-632.

Kylander, M. E., Ampel, L., Wohlfarth, B., & Veres, D. (2011). High‐resolution X‐ray fluorescence core scanning analysis of Les Echets (France) sedimentary sequence: new insights from chemical proxies. *Journal of Quaternary Science, 26*(1), 109-117.

Lamberton, C. (1934). *... Contribution à la connaissance de la faune subfossile de Madagascar*: Imprimerie moderne de l'Emyrne, G. Pitot & cie.

Last, J. (1895). Notes on Western Madagascar and the Antinosi country. *The Geographical Journal, 6*(3), 227-252.

Lohse, J. C., Culleton, B. J., Black, S. L., & Kennett, D. J. (2014). A Precise Chronology of Middle to Late Holocene Bison Exploitation in the Far Southern Great Plains. *Journal of Texas Archaeology and History, 1*, 94-126.

MacPhee, R., & Raholimavo, E. M. (1988). Modified subfossil aye-aye incisors from southwestern Madagascar: species allocation and paleoecological significance. *Folia Primatologica, 51*(2-3), 126-142.

MacPhee, R. D., & Burney, D. A. (1991). Dating of modified femora of extinct dwarf Hippopotamus from southern Madagascar: implications for constraining human colonization and vertebrate extinction events. *Journal of Archaeological Science, 18*(6), 695-706.

Marion, G., Moreno, J., & Oechel, W. (1991). Fire severity, ash deposition, and clipping effects on soil nutrients in chaparral. *Soil Science Society of America Journal, 55*(1), 235-240.

Mooney, S., & Radford, K. (2001). A simple and fast method for the quantification of macroscopic charcoal from sediments. *Quaternary Australasia, 19*(1), 43-46.

Omran, E.-S. E. (2016). A simple model for rapid gypsum determination in arid soils. *Modeling Earth Systems and Environment, 2*(4), 1-12.

Ramsey, C. B. (2009). Bayesian analysis of radiocarbon dates. *Radiocarbon, 51*(1), 337-360.

Ripley, B., Venables, B., Bates, D. M., Hornik, K., Gebhardt, A., Firth, D., & Ripley, M. B. (2013). Package ‘mass’. *Cran r, 538*, 113-120.

Roig, J., Tucker, R., Delor, C., Peters, S., & Théveniaut, H. (2012). Carte Géologique de la République de Madagascar à 1/1,000,000. *Ministère des Mines, PGRM, Antananarivo, République de Madagascar, 1*(1,000,000).

Salehi, M., Beni, O. H., Harchegani, H. B., Borujeni, I. E., & Motaghian, H. (2011). Refining soil organic matter determination by loss-on-ignition. *Pedosphere, 21*(4), 473-482.

Stafford, T. W., Brendel, K., & Duhamel, R. C. (1988). Radiocarbon, 13 C and 15 N analysis of fossil bone: removal of humates with XAD-2 resin. *Geochimica et Cosmochimica Acta, 52*(9), 2257-2267.

Stafford, T. W., Hare, P. E., Currie, L., Jull, A. T., & Donahue, D. J. (1991). Accelerator radiocarbon dating at the molecular level. *Journal of Archaeological Science, 18*(1), 35-72.

Stuenes, S. (1989). Taxonomy, habits, and relationships of the subfossil Madagascan hippopotami Hippopotamus lemerlei and H. madagascariensis. *Journal of Vertebrate paleontology, 9*(3), 241-268.

Tattersall, I. (1987). Itampolo: two subfossil sites in Madagascar. *Journal of Vertebrate paleontology, 7*(3), 342-343.

Team, R. C. (2013). R: A language and environment for statistical computing.

Teviotdale, D. (1929). Notes on stone and moa-bone fish-hook shanks in the Otago University Museum. *The Journal of the Polynesian Society, 38*(4 (152), 270-280.

Tuross, N., Fogel, M. L., & Hare, P. (1988). Variability in the preservation of the isotopic composition of collagen from fossil bone. *Geochimica et Cosmochimica Acta, 52*(4), 929-935.

Vallet-Coulomb, C., Gasse, F., Robison, L., Ferry, L., Van Campo, E., & Chalié, F. (2006). Hydrological modeling of tropical closed Lake Ihotry (SW Madagascar): Sensitivity analysis and implications for paleohydrological reconstructions over the past 4000 years. *Journal of Hydrology, 331*(1), 257-271.

van Geel, B., Gelorini, V., Lyaruu, A., Aptroot, A., Rucina, S., Marchant, R., . . . Verschuren, D. (2011). Diversity and ecology of tropical African fungal spores from a 25,000-year palaeoenvironmental record in southeastern Kenya. *Review of Palaeobotany and Palynology, 164*(3-4), 174-190.

Van Klinken, G. J. (1999). Bone collagen quality indicators for palaeodietary and radiocarbon measurements. *Journal of Archaeological Science, 26*(6), 687-695.

Vérin, P., & Battistini, R. (1971). Les anciens habitats de Rezoky et d'Asambalahy. *Taloha, 4*, 29-49.

Virah-Sawmy, M., Gillson, L., Gardner, C. J., Anderson, A., Clark, G., & Haberle, S. (2016). A landscape vulnerability framework for identifying integrated conservation and adaptation pathways to climate change: the case of Madagascar’s spiny forest. *Landscape Ecology, 31*(3), 637-654.

Wang, L., Brook, G. A., Burney, D. A., Voarintsoa, N. R. G., Liang, F., Cheng, H., & Edwards, R. L. (2019). The African Humid Period, rapid climate change events, the timing of human colonization, and megafaunal extinctions in Madagascar during the Holocene: Evidence from a 2m Anjohibe Cave stalagmite. *Quaternary Science Reviews, 210*, 136-153.

White, E. I. (1930). Fossil hunting in Madagascar. *Natural History Magazine, 2*(15), 209-235.
